# Bitter peptide prediction using graph neural networks

**DOI:** 10.1186/s13321-024-00909-x

**Published:** 2024-10-07

**Authors:** Prashant Srivastava, Alexandra Steuer, Francesco Ferri, Alessandro Nicoli, Kristian Schultz, Saptarshi Bej, Antonella Di Pizio, Olaf Wolkenhauer

**Affiliations:** 1 Institute of Computer Science, University of Rostock, 18051 Rostock, Germany; 2 Section III In Silico Biology & Machine Learning, Leibniz Institute for Food Systems Biology at the Technical University of Munich, 85354 Freising, Germany; 3 Professorship for Chemoinformatics and Protein Modelling, TUM School of Life Sciences, Technical University of Munich, 85354 Freising, Germany; 4 Indian Institute of Science Education and Research Thiruvananthapuram, Maruthamala P. O, Vithura, 695551 Kerala India

**Keywords:** Peptides, Representation learning, Bitter taste, Food

## Abstract

Bitter taste is an unpleasant taste modality that affects food consumption. Bitter peptides are generated during enzymatic processes that produce functional, bioactive protein hydrolysates or during the aging process of fermented products such as cheese, soybean protein, and wine. Understanding the underlying peptide sequences responsible for bitter taste can pave the way for more efficient identification of these peptides. This paper presents BitterPep-GCN, a feature-agnostic graph convolution network for bitter peptide prediction. The graph-based model learns the embedding of amino acids in the bitter peptide sequences and uses mixed pooling for bitter classification. BitterPep-GCN was benchmarked using BTP640, a publicly available bitter peptide dataset. The latent peptide embeddings generated by the trained model were used to analyze the activity of sequence motifs responsible for the bitter taste of the peptides. Particularly, we calculated the activity for individual amino acids and dipeptide, tripeptide, and tetrapeptide sequence motifs present in the peptides. Our analyses pinpoint specific amino acids, such as F, G, P, and R, as well as sequence motifs, notably tripeptide and tetrapeptide motifs containing FF, as key bitter signatures in peptides. This work not only provides a new predictor of bitter taste for a more efficient identification of bitter peptides in various food products but also gives a hint into the molecular basis of bitterness.

**Scientific Contribution**

Our work provides the first application of Graph Neural Networks for the prediction of peptide bitter taste. The best-developed model, BitterPep-GCN, learns the embedding of amino acids in the bitter peptide sequences and uses mixed pooling for bitter classification. The embeddings were used to analyze the sequence motifs responsible for the bitter taste.

**Supplementary Information:**

The online version contains supplementary material available at 10.1186/s13321-024-00909-x.

## Introduction

Peptides, historically defined as polypeptides having 2-50 amino acids, play critical roles in human physiology, acting as hormones, neurotransmitters, and growth factors, and, therefore, they have contributed significantly to the advancement of biological and chemical science [1, 2]. The overall activity of a peptide is largely encoded and thus dependent upon the properties of the amino acids at specific sites within the chain. However, Quantitative Structure-Activity Relationship (QSAR) methods, developed to rationalize and predict the activity of molecules based on their structures, are more challenging when applied to peptides. Indeed, changing the amino acid at a site potentially changes the way it folds.

Deep-learning algorithms have recently shown promising results in QSAR applications [3–5] and decoding the protein/peptide language too [6, 7]. In this article, we present a deep-learning predictor of peptide bitter taste. Bitter taste highly affects food preference and drug compliance [8]. Moreover, bitter taste receptors expressed in extra-oral tissues are found to be involved in pathological disorders and suggested as potential drug targets [9, 10]. Therefore, the prediction of bitter taste has attracted growing interest, and machine-learning predictors were proposed, including classifiers of bitter peptides [11]. Moreover, bitter peptides have been used since the first developments of peptide QSAR models and represent a valuable dataset for testing the applicability and performance of new methods [12].

Predictors of peptide bitter taste were developed in the group of Shoombuatong, using the BTP640 dataset of bitter and non-bitter peptides [13–15]. The first predictor, iBitter-SCM, is a sequence-based predictor that uses the scoring card method (SCM) [13]. Initial dipeptide propensity scores are computed and then refined via a genetic algorithm to create augmented dipeptide propensity scores and amino acid propensity scores, which are used for the final classification. Bert4Bitter is instead based on natural language processing. Peptide sequences are translated into word vectors, and the importance of amino acids is computed (into a vector n-D) via the term frequency-inverse document frequency method. Compared to convolutional neural networks and short-term memory neural networks, the BERT-based model turned out to be more efficient and robust. Another recent predictor, iBitter-Fuse, uses support vector machine [15]. Different methods are used to encode the peptide features: dipeptide composition, amino acid composition, pseudo amino acid composition, amphiphilic pseudo amino acid composition, and physicochemical properties from AAindex. The final model is based on 36 selected features. In 2022, Jiang et al. developed the iBitter-DRLF (Deep Representation Learning Features) predictor [16]. They primarily used deep-learning to determine the best feature performance reached by unified representation and bidirectional long-short-term memory. Secondly, they tested three different algorithms, among which the Light Gradient Boosting Machine showed the best performance. The latest developed bitter predictor is Bitter-RF, a 10-feature composition that uses Random Forest for the prediction [17]. In Table 1, we compiled the performance metrics of existing best performing models, all of which demonstrate very good predictive capabilities.

**Table 1:** Comparison within the independent set validation regarding sensitivity (Sn), specificity (Sp), accuracy (ACC), Matthews coefficient correlation (MCC), area under the curve (AUC) values of best performing bitter peptide predictors that made use of the BTP640 dataset

Tool	AUC [%]	Sn [%]	Sp [%]	ACC [%]	MCC [%]	Website
iBitter-SCM	90	84	84	84	69	iBitter-SCM
BERT4Bitter	96	94	91	92	84	BERT4Bitter
iBitter-Fuse	93	94	92	93	86	iBitter-Fuse
iBitter-DRLF	98	92	98	94	89	iBitter-DRLF
Bitter-RF	98	94	94	94	88	No webserver available

The motivation for our work was to demonstrate the prospect of using Graph Neural Networks for bitter peptide classification, considering that this method (i) does not require feature engineering; (ii) can be more effective on larger datasets if they appear; (iii) offers the advantage of performing inference/explainability-based studies based on the latent embedding generated by the trained model; (iv) provides the possibility of implementing precisely the molecular structure of the peptides (graph-based data).

## Methods

### Data representation

The BTP640 bitter peptide dataset was generated by Charoenkwan [13] and comprises 320 experimentally confirmed bitter peptide sequences and 320 non-bitter peptides (Fig. S1). Peptides in the BTP640 dataset have different lengths, from dipeptides to polypeptides of 39 amino acids, and contain 20 different amino acids (Table S1). The bitter peptides were selected from literature with the prerequisite that their bitter taste was experimentally validated, but the non-bitter peptides were sampled from the BIOPEP database and were not experimentally proven to be non-bitter. Bitter peptides are labeled as ‘Positive’ and non-bitter peptides are labeled as ‘Negative’.

Amino acids in the peptides are considered as nodes *N*, and bonds between them as edges *E* so that peptides can be represented as graphs *G*(*N*, *E*), where $|N| = L$ is the total number of amino acids in the peptide. A peptide graph *G* is assumed to be a simple path graph *P* with length $L-1$ and has a sequence of nodes $P=(n_1, \dots , n_L)$ where, $n_i \in N$ $\forall$ $1 \le i < L$ such that $(n_i, n_{i+1}) \in E$ is an undirected edge between two nodes in *G* [18]. We used a One-Hot encoding scheme to represent the different amino acids. One-Hot encoder transforms categorical data (with $\kappa$ categories) into sparse vectors having length $\kappa$, that represent all categories as binary values. For representing the different node types, we used One Hot encoding and created a one-to-one mapping of amino acids to a 2d vector such that, all the amino acid vectors have a dimension of $1\times 20$. For any peptide graph having *L* amino acids, the node features are represented by a 2d vector with dimension $20 \times L$. Each of the 20 elements of the feature vector represents one type of amino acid out of the 20 amino acids that are present in the BTP640 dataset. For example, the node features of the tripeptide ’DWA’ can be represented as 2d vector dimensions 20 $\times$ 3 where the second dimension of the matrix represents the D, W, and A amino acids.

### BitterPep-GNN models

The goal of GNN is to learn a *k*-dimensional embedding of the node containing the information of both the node and its neighbours. For instance, the node feature of tripeptide ’DWA’ with dimension $20\times 3$ will be transformed to an embedding with dimension $k\times 3$. These node embeddings can be used for various downstream tasks such as node or graph classification, edge labeling, etc. For the case of bitter peptide classification, the transformed *k*-dimensional node embedding is pooled to generate a graph-level embedding. It is worth noticing that in the previous example, regardless of the length of the peptide, the graph embedding will have a dimension of $k\times 1$.

GNN is a stack of node-level hidden layers followed by Multi-Layer Perceptrons (MLP) such that the node embedding of each layer is calculated by aggregating the information of its neighbouring nodes from the previous layer. The learned node embedding is then used for upstream tasks such as graph classification or node classification. Computationally, a GNN model has two components: propagation and pooling. The propagation component is the step where the node information, representing the individual amino acids, is passed on to the subsequent layers, e.g. using a convolution operator. The pooling component is the aggregation operation, where the last node layer is linearly pooled to obtain the graph embedding representing the peptides.

BitterPep-GNN is a graph classification network that predicts the bitter taste of peptides by learning the embedding of amino acids in the form of latent node embedding. We used three different types of GNNs, Graph Convolutional Network (GCN) and Graph Attention Network (GAT), both based on convolutional layers and GraphSAGE, to generate the node-level embeddings, and two pooling techniques for graph embeddings as described below. The input of GNNs is a peptide graph in which the amino acids are represented by nodes present in the graph. The different amino acids, i.e. the nodes, are represented by one-hot encoding.

In a Graph Convolution Network (GCN) layer, the node features are updated by the weighted information of its immediate neighbouring nodes from the previous layer [19]. The update rule for the nodes of the hidden layers is given as:

1 $$\begin{aligned} X^{\text {GCN}}_{i,h} = \sigma \Big [ \sum _{j \in \text {Neb}(i)} p_{ij} W_{h-1} X^{\text {GCN}}_{j,h-1}\Big ], \end{aligned}$$

where, $X^{\text {GCN}}_{i,h}$ is the hidden feature of node $n_i$ in hidden layer *h*, $W_{h-1}$ are the trainable weights, $\text {Neb}$ is the neighbourhood function, and $\sigma$ is the ‘softmax’ activation function. For any node $n_i \in N$ and its neighbour node $n_j \in N$, $p_{ij}$, node normalization factor, is defined as:

2 $$\begin{aligned} p_{ij} = \frac{1}{\sqrt{|\text {Neb}(n_i)| |\text {Neb}(n_j)|}} . \end{aligned}$$

Intuitively, they represent how important the hidden node features $n_j$ are for creating the hidden representation of node $n_i$ in the subsequent layer. Since the coefficient $p_{ij}$ is based on the degree of the nodes $[n_i, n_j]$, they depend on the structure of the graph. This approach of convolution is derived from the spectral domain of graphs [20].

Graph Attention Network (GAT) follows a self-attention-based strategy to propagate the neighbouring node. The attention mechanism attends to all the neighbouring nodes of a node using a linear MLP. The propagation rule can be written as

3 $$\begin{aligned} X^{\text {GAT}}_{i,h} = \sigma \Big [ \sum _{j \in \text {Neb}(i)} a_{ij} W_{h-1} X^{\text {GAT}}_{j,h-1}\Big ], \end{aligned}$$

where, $X^{GAT}_{i,h}$ is the feature vector of $n_i$ in layer *h* and $a_{ij}$ is the attention coefficient of node $n_i$ and $n_j$. The attention coefficients between two nodes are calculated by using the ‘Softmax’ operation ($\sigma$) on the self-attention weights of nodes with their neighbours. Letting self-attention weights be $\alpha _{ij} = \text {Attention}(n_i, n_j)$ [21], the attention coefficient $a_{ij}$ is calculated by

4 $$\begin{aligned} a_{ij} = \frac{\alpha _{ij}}{\sum _{k\in \text {Neb}(i)} \alpha _{ij}}. \end{aligned}$$

In this approach, the attention mechanism coefficients are only dependent on node features and not the structure of the graph. Multi-head attentions (MHA) are also used for enhancing the performance of the network model. For a multi-head attention with *K* attention heads, the GAT layer [22] becomes

5 $$\begin{aligned} X^{\text {GAT}}_{i,h} = \sigma \Big [ \sum _{k=1}^{K} \sum _{j \in \text {Neb}(i)} a^k_{ij} W_{h-1} X^{\text {GAT}}_{j,h-1}\Big ]. \end{aligned}$$

GraphSage uses sampling techniques to learn the latent features of the nodes. In contrast, most GNNs, including GCNs, calculate the node feature vectors based on their entire neighbourhood. There are two steps in GraphSage [23]: sampling and aggregation. In the sampling step, neighbouring nodes are uniformly selected from different edge distances. For any distance *k* with $k \in {1,\dots ,K}$, the update rules are given as,

6 $$\begin{aligned} X_{i,h,\text {Neb}(i)}^{k, \text {GraphSage}} = \text {Agg}_k\bigg (\{X^{k-1}_{j,h-1}, \forall j \in \text {Neb}(i)\}\bigg ). \end{aligned}$$

Each node *i* aggregates the representations of the neighbouring nodes in a separate vector $X_{j, h, Neb(i)}$. The node feature is then calculated as

7 $$\begin{aligned} X_{i,h,\text {Neb}(i)}^{k, \text {GraphSage}} = \sigma \Big [ W^{k}_{h-1}\text {Concat}(X^{k-1}_{i,h-1}, X_{i,h,\text {Neb}(i)}^{k, \text {GraphSage}}) \Big ]. \end{aligned}$$

The sampling technique of GraphSage makes it computationally efficient as compared to the GNNs (which sample the complete neighbourhood). It is also worth noticing that the aggregation function in GraphSage is trainable, making it work efficiently with changing or dynamic graphs.

### Architecture of the BitterPep-GNN models

The architecture of the BitterPep-GNN models has a node embedding module for learning the latent properties of its constituent amino acids and a pooling module for extracting the peptide graph-level embedding and predicting the bitter taste of the peptides. For the node embedding modules, we created a stack of three hidden layers for all the above-mentioned graph networks (GCN, GAT, GraphSAGE). Let $H=\{H_1, H_2, H_3\}$ be the set of hidden layers such that $|H|=3$. Given an input peptide ${\mathcal {P}}$ of with *L* amino acids, with node feature matrix $I_{{\mathcal {P}}}$ with dimension $20 \times L$. The first layer $H_1$ transforms the input amino-acid features $I_{{\mathcal {P}}}$ from the 20-dimensional One-Hot encoded vectors to a $D_f = 16$ dimensional vector. The $H_1$ node features are forwarded to the subsequent layers $H_2$, $H_3$ using ReLU non-linear activation. The equation of the Rectified Linear Unit ReLU operator is defined as:

8 $$\begin{aligned} \text {ReLU}(x) = \text {max}(0,x). \end{aligned}$$

The output $X_{i,H}$ of $H_3$ is the final embedding of all the amino acids in the peptide having shape $(D_{f},L)$. The output amino acid node features were flattened in the pooling module to get the peptide graph vectors with length $D_f$, allowing us to obtain fixed-size vectors, which were then used for downstream classification layers.

We used two pooling schemes to generate the graph-level embedding. The pooling layer takes as input the node level amino acid features $X_{i,3}$ of $H_3$ having node features of dimension $D_f$ and creates a one-dimensional graph-level peptide vector. We used two types of pooling module schemes: meanpool and mixedpool. In the meanpool module, the output embedding $X_3$ is simply averaged to obtain a fixed-size vector $G^{mean}_p$. The meanpool module is defined as:

9 $$\begin{aligned} G^{\text {mean}}_{p} = \frac{1}{L} \sum _{1 \le n \le L} X_{n,H}. \end{aligned}$$

In the meanpool module, the flattened vector $G^{mean}_p$ represents all the amino acids with uniform weight. However, in the mixedpool module scheme, the $X_3$ embedding was flattened by three operations: mean, max, and add. First, the three types of flattened vectors were obtained from $X_3$. Then, the three vectors were added together to obtain the mixedpool output. The mixedpool module can be defined as:

10 $$\begin{aligned} G^{\text {mix}}_{p} = \frac{(L+1)}{L} \sum ^L_{n=1} X_{n,H} + \text {max}^L_{n=1} [X_{n,H}]. \end{aligned}$$

Both of the pooling modules provide a representation of the peptide based on its amino acids. The peptide vector output of the pooling layer with shape $(D_f)$ is then transmitted to the subsequent layer with a dropout probability of $P_d$ [24]. The final layer is the output layer *O*, which is a linear classifier.

11 $$\begin{aligned} O_p = W_{\text {Lin}} \text {Dropout}_{P_d}(G_p) \end{aligned}$$

where $W_{\text {Lin}}$ are the trainable weights of the classification layer.

We combined the three embedding types and the two pooling schemes to create six graph networks: BitterPep-GCN-Meanpool, BitterPep-GCN-Mixedpool, BitterPep-GAT-Meanpool, BitterPep-GAT-Mixedpool, BitterPep-Sage-Meanpool, and BitterPep-Sage-Mixedpool. The parameters of BitterGNNs were fixed for all networks. The GNN module has three node embedding hidden layers $|H| = 3$ that were stacked, with each layer having $D_f = 16$ dimensional node embedding. In the pooling module, the dropout probability was set to $d = 0.1$.

The models were subjected to a benchmarking process using the BTP640 dataset and a 10-fold cross-validation strategy. The dataset was divided into ten subsets, each containing 64 peptides that were randomly selected without replacement. For any run *r* such that $1 \le r \le 10$, in 10-fold validation, the models were trained on the 576 peptides that were not present in the $r^{th}$ fold of the dataset. During the evaluation, the 64 peptides present in the $r^{th}$ fold were used. The accuracy and ROC-AUC were calculated for each of the 10 runs for all the models. We used fixed random seeds for splitting the data to ensure reproducibility. We compared the performance of the models using the average accuracy and ROC values recorded over the 10-fold cross-validation and the accuracy and ROC values of the top-performing fold in the 10-fold cross-validation run.

### Interpretation methods

The best performing model was analysed to understand the features responsible for the bitter peptide prediction. To facilitate visualisation and interpretation of the data, the peptide embeddings were transformed into a 2D array using t-SNE and then clustered using K-means. We acknowledge the availability and debate over various dimensional reduction and clustering methods [25–27]. However, since this analysis was not intended for prediction or decision-making, we chose methods that provided the clearest representation of our data.

To analyse the importance of individual amino acid and peptide motifs, we extracted the amino acid embedding, and we generated normalized importance scores (Table S6). The importance scores were calculated by averaging the output $X_3$ of all the occurrences of a substructure in the peptides. For any substructure having length $l_{s}$ and occurring $O_{s}$ times in the peptides, the importance score $I_{s}$ can be calculated as:

12 $$\begin{aligned} I_{s} = \frac{1}{O_{s}} \sum _{1<i}^{O_s}{\Big [\frac{1}{l_s} \sum _{1<i}^{l_{s}}{X_{s,i,3}} \Big ] } \end{aligned}$$

where $X_{s,i,3}$ is the output of $i^{th}$ amino acid in a substructure *s*. A total of 927 substructures, comprising amino acids with a length of one to four, were identified in the bitter peptide set. In the non-bitter peptide set, 1315 substructures were found to have a length between one and four amino acids.

For decoding the relevance of the amino acid contribution to peptides’ bitter taste, we applied Gradient Class Activation Mapping (Grad-CAM), a variation of the Class Activation Mapping (CAM) method, providing visual explanations for decisions made by any convolutional network-based model, regardless of their layer architecture [28, 29]. It exploits the gradients flowing into the last convolutional layer to highlight which graph nodes are most important for the model‘s decision during the classification task. We define the *k* th graph convolutional feature map at layer *l* as:

13 $$\begin{aligned} F^{l}_{k}(X,A) = \sigma (VF^{l-1}(X,A)W^{k}_{l}) \end{aligned}$$

with *A* being the adjacency matrix, *X* the node attributes, $W^l_k$ the *k*-th column of $W^l$ trainable convolutional weights, $\sigma$ the activation function, while $F^l_{k_,n}$ represents the *k*-th feature for the n node at the *l*-th layer [30]. Let ${\hat{D}}_{ii}= \sum _{j}{\hat{A}}_{ij},{\hat{A}}=A + I_N$ with $I_N$ being the added self connections, then *V* is calculated as $V={\hat{D}}^{\frac{-1}{2}}{\hat{A}}{\hat{D}}^{\frac{-1}{2}}$. The global mean pooling feature after the last convolutional layer *l* is computed with the formula:

14 $$\begin{aligned} e_k=\frac{1}{N} \sum ^N_{n=1} F^l_{k,n}(X,A) \end{aligned}$$

with the class score $y^e=\sum _{k} w^C_k e_k$. The Grad-CAM weights are therefore defined as:

15 $$\begin{aligned} \alpha ^{i,C}_k = \frac{1}{N} \sum ^N_{n=1} \frac{\delta y^C}{\delta F^l_{k,n}} \end{aligned}$$

Finally, the neat map from the layer *l* is calculated as:

16 $$L_{{{\text{Grad - CAM}}}}^{C} [l,n] = {\text{ReLU}}\,\left( {\sum\limits_{k} {\alpha _{k}^{{l,C}} } F_{{k,n}}^{l} \left( {X,A} \right)} \right)$$

## Results and discussion

### BitterPep-GNN

Graph-based deep learning methods are a type of neural network that is used for data with non-Euclidean geometries such as graphs [20]. The non-regularity of data structures led to the massive success of Graph Neural Networks. In natural language processing, GNN models are used to generate word and document embedding for text corpus for tasks like text classification. Text elements are represented by a line graph, and the relationship between these elements is embedded in the graph via links between nodes.

A peptide can be considered as a text sequence. Amino acids in a peptide are represented as nodes, and bonds between them as edges. Therefore, the problem of bitter peptide prediction can be addressed as a graph classification problem. GNN models then are focused on combining information of nodes and their neighbour nodes [31, 32].

While GNNs have been successfully developed for predicting biological activity or properties like absorption, distribution, excretion, metabolism, and toxicity (ADMET) of drug candidates [32–34], only a few studies also explored the potential of developing prediction models of peptides via GCN [35, 36].

We tested six BitterPep-GNN models, the results of the cross-validation-based benchmarking study are given in Table 2. The developed models do not outperform the existing methods the existing methods (Table 1), but achieve comparable results.

**Fig. 1:**
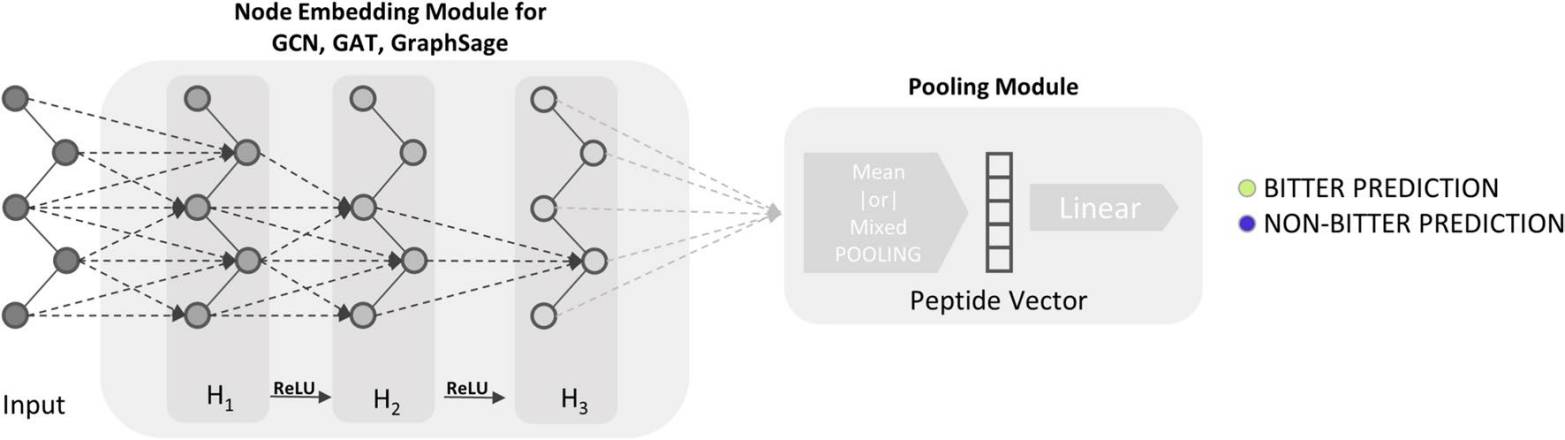
Schematic representation of the model architecture. Each amino acid present in the input peptides is processed by the three graph neural layers. The output of the graph neural networks is pooled to obtain a fixed-size vector. At last, the fixed-size vector is used by a linear classifier layer

Our models were generated by combining three types of graph neural networks and two types of pooling modules. The architecture of the models is given in Fig. 1. The GCN Embedding proved to have the highest accuracy. The BitterPep-GCN model showed superior performance than BitterPep-GAT and Sage networks, regardless of the pooling modules. Among the three GNNs, BitterPep-GCN network has the highest average 10-fold accuracy value, and the highest top fold accuracy and top fold ROC values. For the average 10-fold ROC value, BitterPep-GAT achieved the best performance.

**Table 2:** 10-fold cross-validation performance of the three GNN embedding types with two types of pooling layers

GNN	Pooling	Avg Acc	Avg ROC	Top Acc	Top ROC
GCN	Mean	0.83	0.88	0.91	0.97
	Mixed	**0.86**	0.89	**0.95**	**0.98**
GAT	Mean	0.81	0.88	0.88	0.93
	Mixed	0.83	**0.90**	0.89	0.97
GraphSage	Mean	0.81	0.88	0.89	0.93
	Mixed	0.83	0.89	0.89	0.94

Highest achieved performance in bold

For the pooling modules, Mixedpool schema showed better 10-fold cross-validation performance than the Meanpool schema when combined with the GNNs. In the case of BitterPep-GCN, the average 10-fold accuracy for the Meanpool and Mixedpool schema are 0.83 and 0.86; the top fold accuracy values are 0.91 and 0.95 for Meanpool and Mixedpool, respectively. Moreover, for the BitterPep-GCN, Mixedpool schema has higher average 10-fold ROC and top fold ROC values than Meanpool schema. For BitterPep-GAT combined with Meanpool schema, the average 10-fold accuracy and top fold accuracy values are 0.81 and 0.88. BitterPep-GAT combined with Mixedpool schema has the average 10-fold accuracy and top fold accuracy of 0.83 and 0.89 showing that Mixedpool has better performance. Similarly, the BitterPep-Sage network combined with the Mixedpool schema has an average 10-fold accuracy of 0.83 and top fold accuracy of 0.89, and average and top fold accuracy values of 0.81 and 0.89 with the Meanpool schema. With regards to the 10-fold cross-validation benchmarking study, BitterGCN-Mixedpool showed the best bitter classification performance among the six BitterPep-GNN models.

### Size, hydrophobicity, and BitterPep-GCN predictions

The relationship between the chemical structure of bitter peptides and their elicited bitterness has been studied and disputed extensively for a long time. Hydrophobicity has been pointed out as a relevant molecular feature for determining bitterness since 1971 when Ney formulated the Q rule to estimate peptide bitter taste [38]. To determine whether and to what extent hydrophobicity is incorporated into our best performing model, BitterPep-GCN, we analysed the output of the embedding module.

**Fig. 2:**
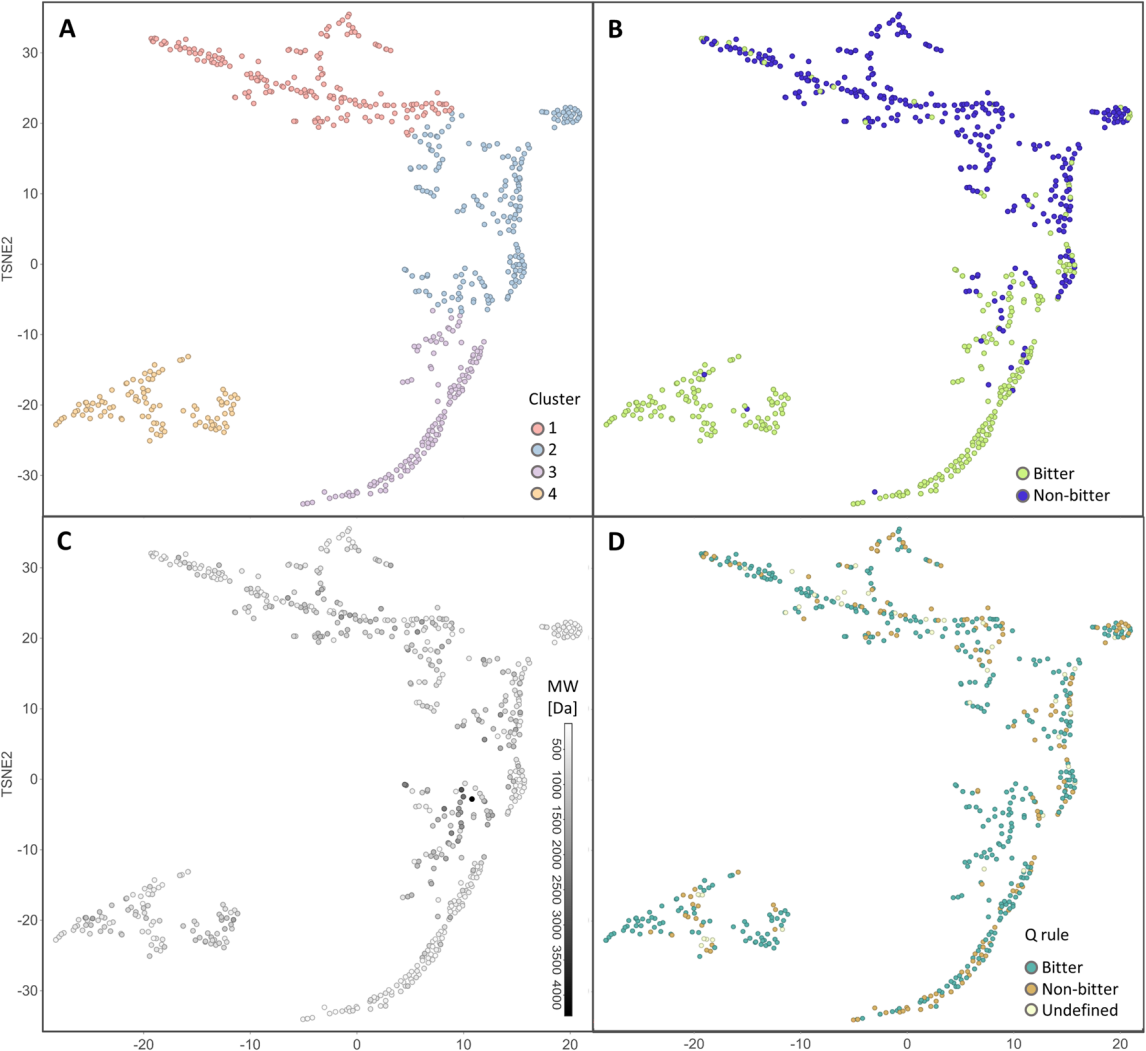
2D t-SNE of peptide graph embedding of the BTP640 dataset using BitterPep-GCN model.** A** The plot is coloured by cluster association: cluster 1 is coloured in coral, cluster 2 is coloured in light blue, cluster 3 is coloured in light violet and cluster 4 is coloured in dark yellow.** B** The plot is coloured by bitterness, as labeled in the BTP640 dataset: lime-green-coloured for bitter peptides and blue-coloured for non-bitter peptides.** C** The plot is coloured in grey scales according to the molecular weight (MW).** D** The plot is coloured by Q values: peptides with Q values above 1400 cal/mol (predicted as bitter for the Q rule) are coloured in green, peptides with Q values below 1300 (predicted as non-bitter for the Q rule) are coloured in brown, or whitish if the Q value is undefined with values in between 1300 and 1400 cal/mol

The initial step in this process was to generate the peptide space of our dataset, which was then used as a visualization tool to navigate among the peptides. We used t-SNE visualization and grouped the peptides into four clusters. The clusters differ in the ratio of bitter and non-bitter peptides and, from cluster 1 to cluster 4, they progressively increase in bitter population (Table S2, Fig. 2A, B). Cluster 4 has the highest percentage of bitter peptides (contains 121 peptides with only 2 non-bitter peptides) and the lowest number of incorrect predictions (Fig. S2), but also visibly different embeddings, so it is isolated from the other three clusters. Cluster 1 is mostly populated by non-bitter peptides (only 16 bitter peptides). The area between clusters 2 and 3 has a mixture of bitter and non-bitter peptides overlaps (Fig. 2B) and is indeed the most challenging for prediction (Fig. S2).

**Fig. 3:**
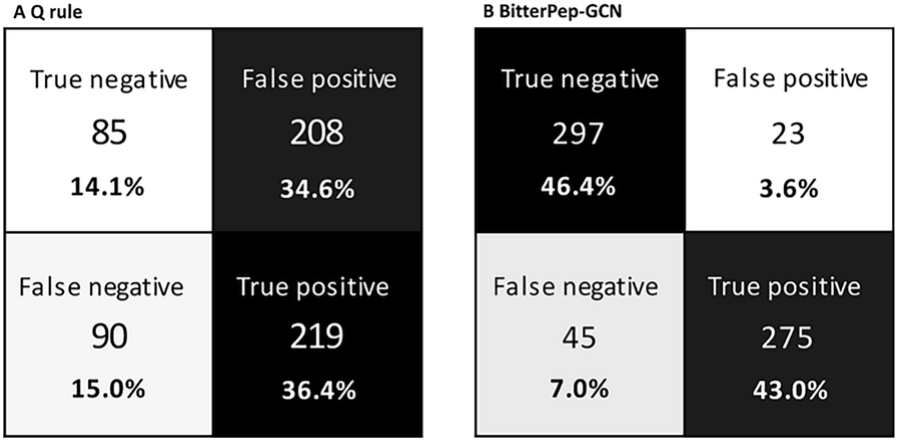
Confusion matrices of the prediction via the Q rule (**A**) and the prediction via BitterPep-GCN (**B**). In the confusion matrix analysis of the Q rule-based prediction, peptides with a Q-value between 1300-1400 cal/mol were not included

Peptides in the four clusters have a wide distribution of the molecular weight (MW), spanning from 500 to 4000. The longest peptides are found within cluster 2 (Fig. 2C, Table S4). We monitored the hydrophobicity of peptides in the four clusters, by mapping the t-SNE with the peptides’ Q values (Fig. 2D). The Q value is the average free energy for the transfer of the amino acid side chains from ethanol to water $(Q = \sum \Delta f/n)$, i.e., as originally applied by Tanford [37], an estimate of the relative hydrophobicity of amino acids and peptides. Ney found that most bitter peptides had Q values $> 1400$ cal/mol, whereas non-bitter peptides had Q values $<1300$ cal/mol [38]. Figure 2D illustrates that the clusters do not differ in hydrophobicity and each of them contains peptides with Q values higher than 1400 cal/mol and lower than 1300 cal/mol. This also suggests a low correlation between the BitterPep-GCN predictions and Q values, as better shown in the confusion matrices (Fig. 3).

BitterPep-GCN shows high rates of true positives (TPR) and true negatives (TNR) and, interestingly, has a higher false negative rate (7%) than a false positive rate (FPR) (3.6%). The Q rule classifies 304 peptides correctly. It is good at recognizing bitter peptides (36.4% TPR), but also shows a high FPR. The Q value shows a comparable number of correctly determined bitter and non-bitter peptides in all clusters (60 out of 166 peptides in cluster 1, 73 out of 183 peptides in cluster 2, 85 out of 138 peptides in cluster 3, 86 out of 111 peptides in cluster 4, Table S3). The high rate of false positive values is located mainly in clusters 1 and 2, and in the area between clusters 2 and 3.

### Amino acid contributions to the BitterPep-GCN predictions

The bitter taste is the result of the interaction of peptides with bitter taste receptors (TAS2Rs) on the tongue. TAS2Rs belong to the G protein-coupled receptor (GPCR) superfamily [39]. Modeling studies of peptides in complex with TAS2Rs and structural investigation of peptides in complex with other GPCRs suggest that TAS2R could recognize core signatures or motifs in peptide sequences in their orthosteric binding site [40–43]. Therefore, some structural motifs may play a significant role in the bitter taste of the entire peptide.

As our model treats peptides as line graphs, to identify signatures carrying bitter information in the peptide sequences, we calculated the activity of amino acids and peptides by aggregating and averaging the embedding of all substructures, and analysed the contribution of individual amino acids to the predictions.

**Fig. 4:**
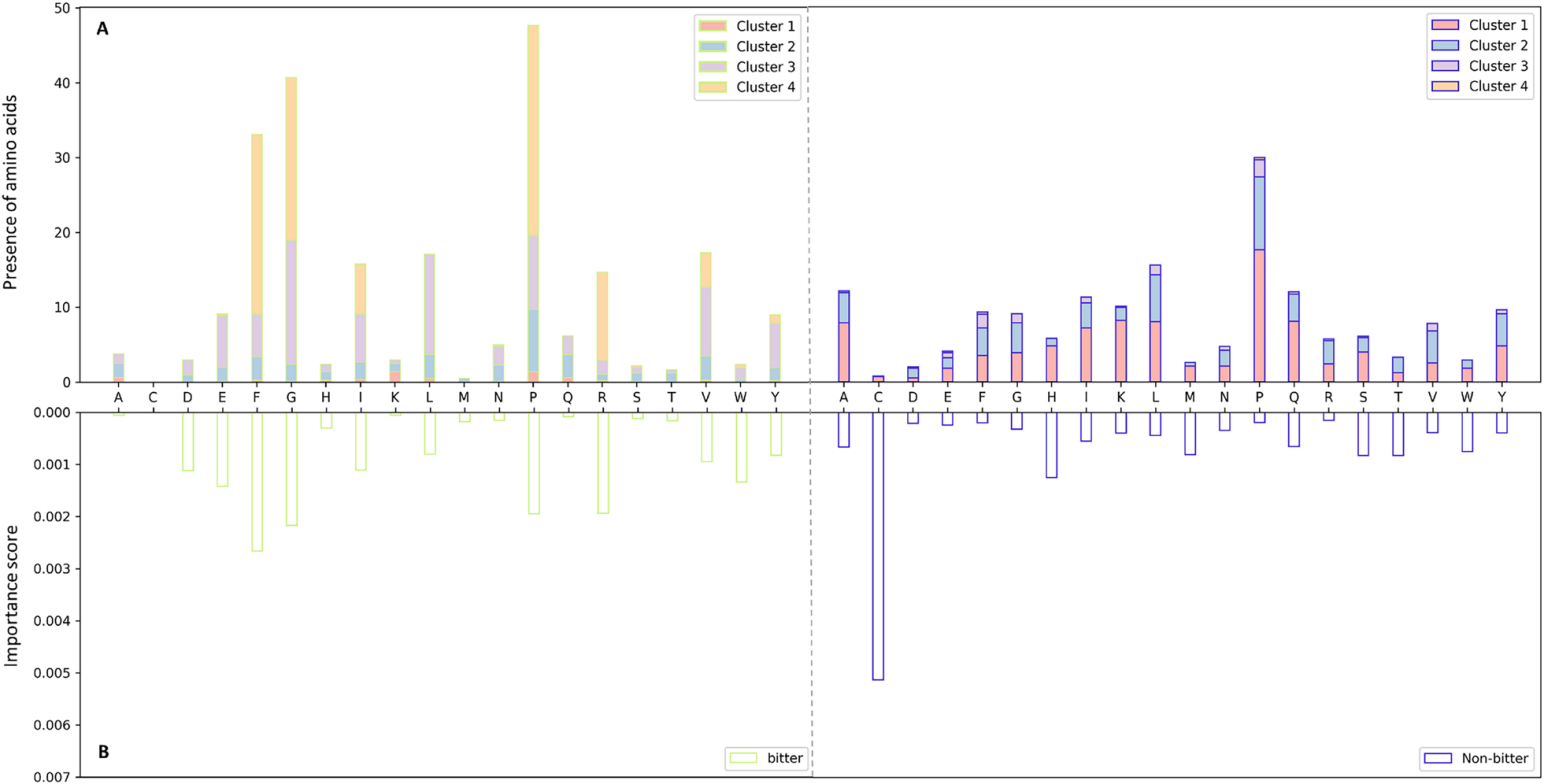
**A** Occurrence of the individual amino acids in the bitter and non-bitter set. Histograms are coloured according to the cluster association.** B** Importance scores of individual amino acids in the bitter and non-bitter sets

In Fig. 4, we report the importance scores (panels on the bottom) and occurrence (panels on the top) of individual amino acids in the bitter (panels on the left) and non-bitter (panels on the right) sets. Amino acids A, C, M, N, Q, S, and T have been identified as having a greater impact for a peptide to be non-bitter. Cysteine is only present among non-bitter peptides and occurs only in cluster one. Interestingly, A, C, M, and Q are not represented in any of the peptides of cluster 4. In contrast, the most prevalent amino acids in this cluster are F, G, P, and R, which exhibit the highest activity for a bitter peptide. The model appears to capture the propensity of specific amino acids for bitter vs. non-bitter prediction. Moreover, this does not seem to correlate with the imbalanced composition of the two sets. While F, G, and R are distributed differently in the bitter and non-bitter sets, P is of greater importance for the bitter predictions despite a similar and high distribution in the two sets. This can be observed also for other amino acids, such as D and E. Another example is the two amino acids V and I, which appear to occur in all clusters and are present in both bitter and non-bitter sets. However, according to the BitterPep-GCN model, they are of greater importance for bitterness. The lack of a correlation between the importance scores and the occurrence of amino acids suggests that the model considers more aspects than just the amino acid composition.

**Fig. 5:**
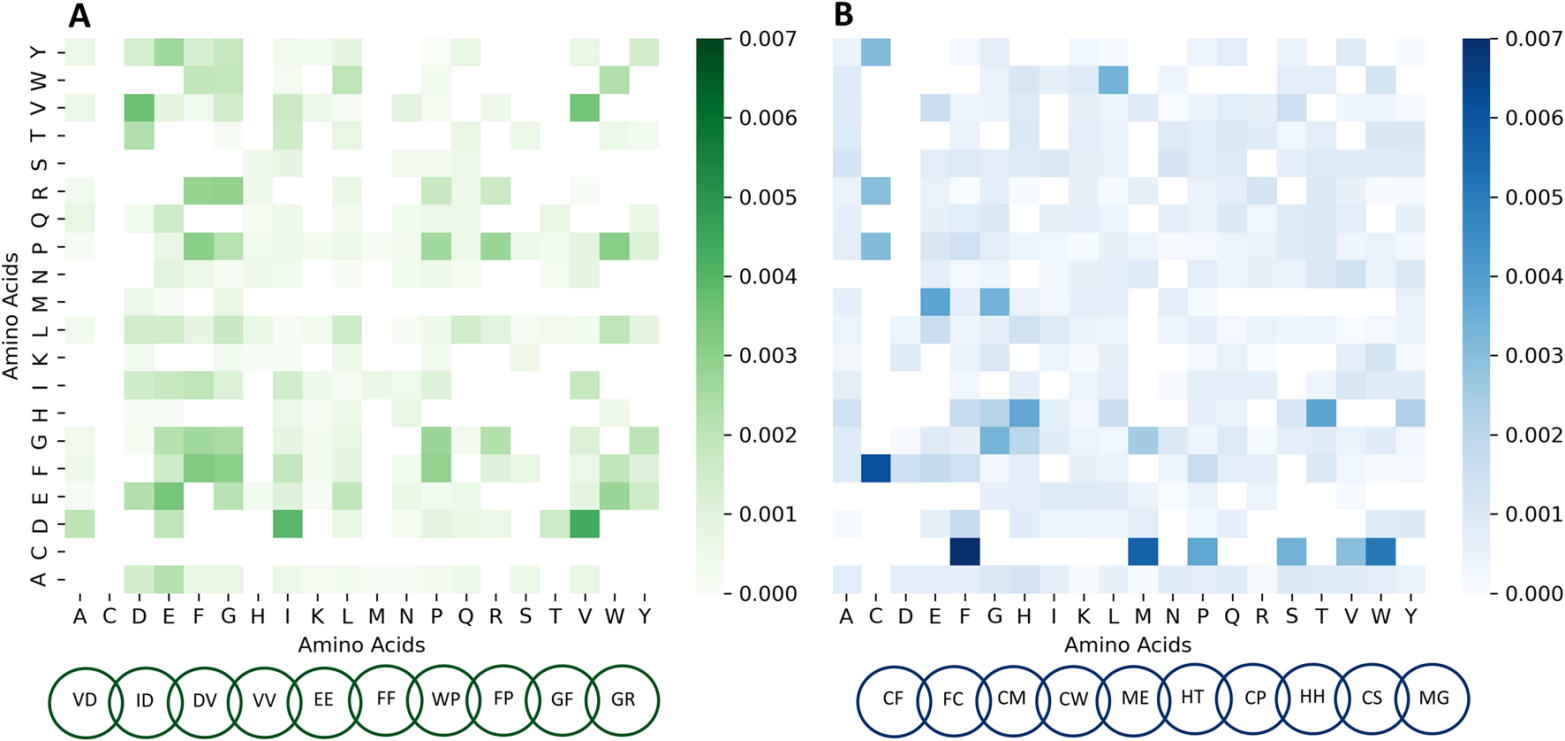
The heatmaps illustrate the importance scores of dipeptide motifs, calculated for** A** bitter taste peptides (shown in green shades) and** B** non-bitter peptides (shown in blue shades)

To evaluate the combination of amino acids in dipeptide motifs, we created heatmaps with all the combinations and the important scores for both bitter (Fig. 5A) and non-bitter (Fig. 5B) activity.

The dipeptide sequences with high scores (scores > 0.0029) are VD, ID, DV, VV, EE, FF, WP, FP, GF and GR, confirming the propensity of specific amino acids for bitter taste. Similarly, among dipeptide motifs that have high importance for non-bitterness, we mostly found C-containing motifs, i.e., CF, FC, CM, CW, and CP. However, different motifs get different scores, for example, CM has a higher score than MC, suggesting that the model also weights differences in the positions of amino acids in the sequence.

### BitterPep-GCN insights on the signature motifs driving peptide bitter taste

As discussed above, the model pinpoints a propensity of specific amino acids for bitter taste predictions. To better characterize bitterness signatures, we analysed the 20 motifs with the highest importance scores among the substructures (individual amino acids, di-, tri- and tetrapeptide sequences) extracted from the embeddings in the bitter peptide set (Table 3).

**Table 3:** Lists of the amino acids, and di-, tri-, and tetrapeptide sequence motifs for the 20 highest importance scores

Amino acids		Dipeptide motifs		Tripeptide motifs		Tetrapeptide motifs	
F	0.00266	VD	0.00437	FPF	0.00443	FPPF	0.00399
G	0.00217	ID	0.00396	PFF	0.00357	PPFG	0.00372
P	0.00194	DV	0.00362	VVV	0.00352	PPFF	0.00368
R	0.00194	VV	0.00351	FPP	0.00350	FFPP	0.00364
E	0.00142	EE	0.00342	FPR	0.00344	FFPG	0.00361
W	0.00134	FF	0.00323	FFP	0.00343	PFFF	0.00358
D	0.00112	WP	0.00310	FPG	0.00342	FPGG	0.00355
I	0.00111	FP	0.00304	RPF	0.00341	GPFF	0.00354
V	0.00095	GF	0.00304	FFF	0.00340	FRPF	0.00346
Y	0.00083	GR	0.00295	FRP	0.00338	PFFG	0.00344
L	0.00081	PF	0.00285	FFR	0.00330	PFFR	0.00339
H	0.00030	FR	0.00284	FFG	0.00320	GRPF	0.00337
M	0.00018	WE	0.00279	PGF	0.00319	FFRP	0.00335
T	0.00016	RP	0.00276	EEE	0.00309	RPFF	0.00335
N	0.00015	PG	0.00276	PGR	0.00304	FFPR	0.00335
S	0.00012	EY	0.00266	GRP	0.00304	PPPF	0.00331
Q	0.00008	PP	0.00263	GPP	0.00302	PFPP	0.00329
A	0.00006	FG	0.00260	FGG	0.00301	FPGR	0.00327
K	0.00006	GG	0.00245	GFF	0.00301	PFPG	0.00324
C	-	WW	0.00228	RPG	0.00298	RPFG	0.00321

As a confirmation of the amino acid propensities, we found that only 10 out of the 20 amino acids represented in the dataset are present in the list of highest-scored motifs. And five of them, i.e., F, G, P, R, and V, were identified as specific amino acids for bitter taste (Fig. 4). However, as the peptide lengths in the datasets are imbalanced towards short peptides (Table S1), it is not necessarily the case that all the motifs with high scores are motifs of peptides in the dataset. Therefore, to identify among these highly scored motifs those that could be the signatures driving the bitter taste to the peptides, we also looked at the occurrence in the dataset. Indeed, VD, ID, and WP are present in the dataset only as dipeptides themselves and do not occur in any other longer peptide in the dataset, and DV is only contained in one longer peptide. The dipeptide EE and the tripeptide EEE occur both exclusively in four bitter peptides of the dataset. The dipeptide VV is bitter itself, but only three out of nine peptides containing this motif are bitter. VVV is also identified as a highly scored motif, and interestingly, it occurs at the beginning or the end of five bitter peptides in the dataset. The dipeptide motif GR is present both in bitter and non-bitter peptides. It results in the two highly scored tripeptide motifs PGR (present in the tetrapeptide motif FPGR) and in GRP that occur in the highly scored tetrapeptide motif GRPF and 7 bitter peptides and one non-bitter peptide of all the set.

However, on the other hand, we have examples of motifs that are highly represented in the bitter peptides. The dipeptide motif GF is also bitter itself and is present in 22 peptides of the dataset, 21 of which are bitter. The dipeptide FF is bitter itself and, as a motif, is present in 33 bitter peptides. The dipeptide motif FF is also present in highly scored tripeptide motifs: PFF (which itself occurs in the tetrapeptide motifs PPFF, PFFF, GPFF, PFFG, PFFR, RPFF, and is overall reported in 14 bitter peptides in the dataset), FFF (which occurs in the tetrapeptide motif PFFF), FFR (which occurs in the tetrapeptide motif PFFR and FFRP), FFG (also in the tetrapeptide motif PFFG) and GFF (which does not occur in one of the 20 highly scored tetrapeptide motif but is present in 9 bitter peptides). Interestingly, the tripeptide motifs FFF, FFR, and GFF are exclusively presented in bitter peptides. The tripeptide motifs PFF and FFG are present mostly in bitter peptides (i.e., in 19 bitter peptides out of the 21 peptides containing them). Tetrapeptide motifs containing the dipeptide FF are bitter themselves or exclusively present in bitter peptides. PF is the most frequent dipeptide motif in the dataset. It occurs 86 out of 98 times in bitter peptides, and it results in the highly scored tripeptide motifs FPF, PFF, and RPF. The tripeptide motif FPF is bitter itself and occurs in 2 non-bitter peptides. The tripeptide motif PFF is present only in one non-bitter peptide (PFFDPQIP) and in 14 bitter peptides. Tetrapeptide motifs containing PFF (i.e., PPFF, PFFF, GPFF, PFFG, PFFR, RPFF) either are bitter themselves or only occur in bitter peptides. The tetrapeptide motifs PPFG, PPPF, and PFPG do not contain a highly scored tripeptide motif but contain within the highly scored dipeptides FG, PF, PG, and PP and occur in 14 bitter peptides. The tripeptide motif RPF and its highly scored tetrapeptide motifs (FRPF, GRPF, RPFG) are exclusively present in bitter peptides.

The dipeptide motif FP is also bitter itself and occurs 51 out of 64 times in bitter peptides of the dataset and 31 times in the tripeptide motifs of bitter peptides (FPF, FPP, FPR, FFP, and FPG). The most frequent tripeptides are FPP (11 times present and occurs in the highly scored tetrapeptide motifs FPPF and in PFPP), FFP (5 times present and occurs in tetrapeptide motifs FFPP, FFPG, and FFPR) and FPG (13 times present in bitter peptides and occurs in FFPG, FPGG, and FPGR). Whereas FPP, FPR, and FFP are exclusively present in bitter peptides, FPF and FPG are found in non-bitter peptides as well (FPFEVFGK, FFVAPFPFEVFGK, LVYPFPGPIPNSLPQNIPP, and MIFPGGPQL).

FF, FP, and PF are, therefore, highly scored and also the most present in the bitter peptides in the BTP640 dataset. It is, therefore, not surprising that these motifs also obtained high dipeptide propensity scores from the iBitter-SCM [13].

**Fig. 6:**
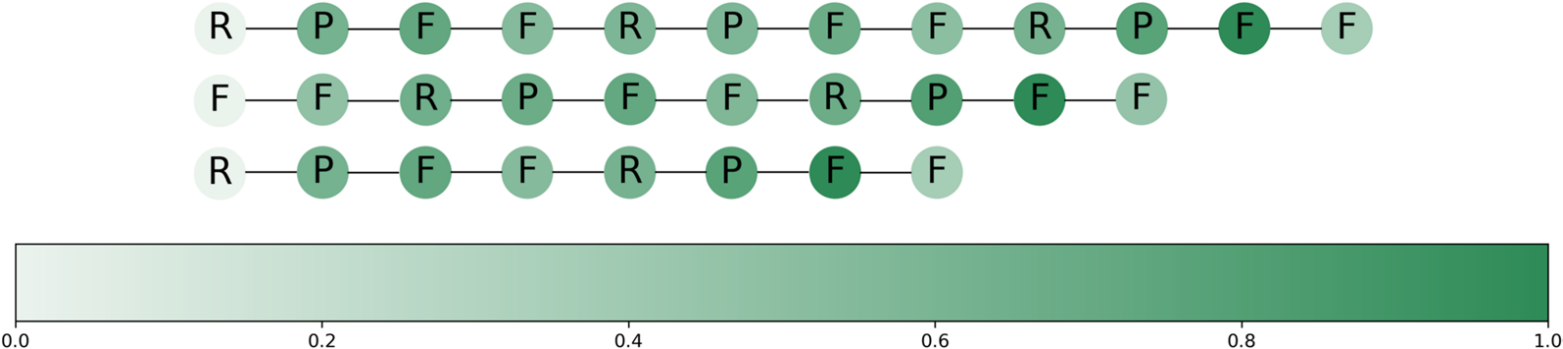
Visualization of the calculated values with Grad-CAM. The importance of the individual amino acid is represented from whitish (for a minor likelihood ) to green (for a greater likelihood) for the development of a bitter taste

The advantages of using GNN in this work rely on being able to capture also the positional relevance of the motifs. GNNs can handle graphs with variable lengths and capture long-range dependencies, which is particularly important in this case, as the peptides in the dataset are different in length and distant residues might have significant interactions. To investigate the individual and positional importance of the amino acids and sequence motifs within the peptides in BitterPep-GCN, we used Grad-CAM (Gradient-weighted Class Activation Mapping). This allowed us to visualize the embedding output of the last convolutional layer to highlight the individual locations for the bitter prediction. We analysed the case of peptides containing the high-scored motifs FF, FFR, and FFRP, i.e. RPFFRPFFRPFF, RPFFRPFF, and FFRPFFRPFF (Fig. 6). All three peptides are correctly predicted to be bitter, but it is evident that the position of amino acids plays a significant role in their contribution to the bitter taste prediction.

## Conclusion

Predictors of bitter peptide taste might have a high impact on the food sector. Considering the constant growth of knowledge of the bitter peptide space and how this will increase the complexity of the systems to be decoded, we believe it is crucial to evaluate novel approaches and develop interpretable models that can illuminate the underlying molecular signature of peptides’ bitter taste, and provide accurate and fast predictions.

In our work, we propose a GNN-based predictive method for bitter peptides that gives way to predicting bitter taste while preserving the underlying amino-acid structure of the peptides. The best-developed model, BitterPep-GCN, learns the embedding of amino acids in the bitter peptide sequences and uses mixed pooling for bitter classification. The model achieved an at-par performance of 10-fold cross-validation compared to previously published models that used the same dataset of peptides. With respect to other predictors, our model offers the advantage that it does not utilize any pre-calculated features or embedding. It only takes into consideration the underlying sequence structure of the peptides and can handle sequences with variable lengths and capture long-range dependencies. Representing the sequence as a graph (even if we started with the linear graph) allows for easier integration of additional structural data in following updates of the predictor. Moreover, considering the graph-based structure of the peptide sequence also adds to the interpretability of the model.

We found that neither the hydrophibicity nor the peptide size can alone explain peptide bitter taste and has no correlation with our model. Indeed, bitterness results from the complex information encoded in the peptide sequence. Therefore, we provided detailed analyses of the BitterPep-GCN predictions aimed at identifying important sequence motifs for peptide bitter taste. The embeddings were used to analyse the sequence motifs responsible for the bitter taste. We generated a list of highly scored motifs (Table 2) and checked for all of them, the occurrence in this pool, and in the dataset in general. This allowed us to capture the correlation between prediction and occurrence, like in the case of the motif FF and tri- and tetrapeptide motifs containing it. The results of the Grad-CAM analysis demonstrate the influence of the motif position on the predictions. Therefore, the model is also capable of capturing structural aspects, which confirms the applicability and advantage of GNN for investigating bitter peptides. However, the current limitation for developing robust predictors is the scarcity of sensory-proofed data, particularly for non-bitter peptides, and the absence of information on taste thresholds. It is therefore recommended that future efforts be directed towards the generation and collection of high-quality data.

## Supplementary Information

Supplementary file 1.

## Data Availability

Publicly available data sets were used for the study. The code is available in the following GitHub repository github.com/srivastavaprashant/bittergnn/. The notebook of the Grad-CAM analysis is available at: https://github.com/dipizio/BitterPep-GCN.
