# Supplementary material for: Bitter peptide prediction using graph neural networks
Source: J Cheminform. 2024 Oct 7;16:111. doi: 10.1186/s13321-024-00909-x (PMC11459932; doi:10.1186/s13321-024-00909-x)
Supplement: Supplementary file 1 [file 13321_2024_909_MOESM1_ESM.docx]

**Supporting Information**

Bitter Peptide Prediction Using Graph Neural Networks

**Prashant Srivastava^1$^, Alexandra Steuer^2,3$^, Francesco Ferri^2^, Alessandro Nicoli^2,3^, Kristian Schultz^1^, Saptarshi Bej^1,4^, Antonella Di Pizio^2,3^*, and Olaf Wolkenhauer^1,3^***

^1^ Institute of Computer Science, University of Rostock, Rostock 18055, Germany

^2^ Leibniz Institute for Food Systems Biology at the Technical University of Munich, 85354 Freising, Germany

^3^ Professorship for Chemoinformatics and Protein Modeling, TUM School of Life Sciences, Technical University of Munich, 85354 Freising, Germany

^4^ IISER Thiruvananthapuram, Maruthamala P. O, Vithura, Kerala, 695551, India.

^$^equal contribution

*Correspondence to a.dipizio.leibniz-lsb@tum.de, olaf.wolkenhauer@uni-rostock.de


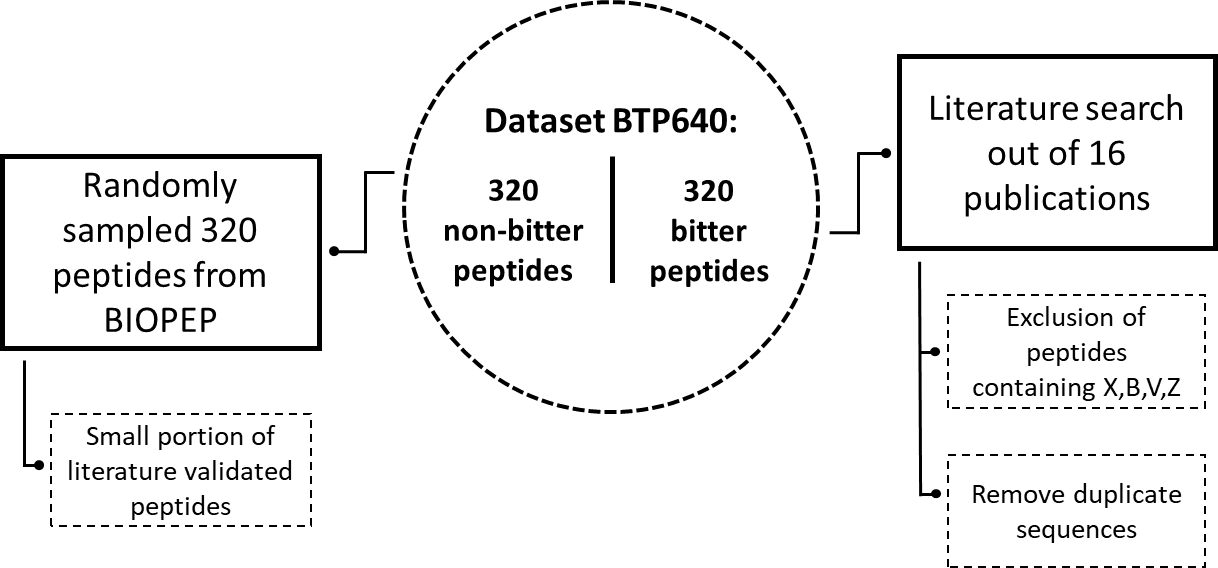
**Figure S1.** BTP640 dataset (Charoenkwan, Yana et al. 2020) composition.

**Figure S2.** T-SNE plot colored by accuracy.


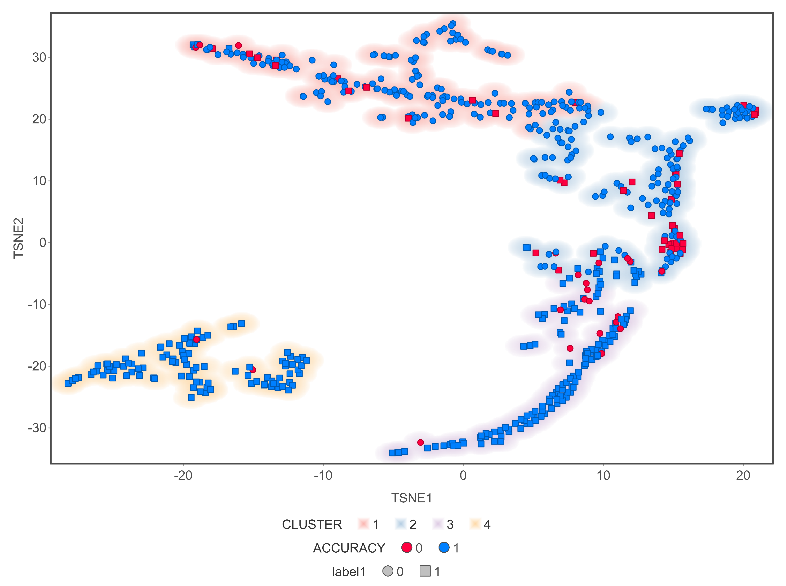


**Table S1.** Length of bitter and non-bitter peptides in the BTP640 dataset

| **BTP640** | **Total number of peptides** | **Number of bitter peptides** | **Number of non-bitter peptides** |
| --- | --- | --- | --- |
| Dipeptides | 137 | 95 | 42 |
| Tripeptides | 146 | 61 | 85 |
| Tetrapeptides | 57 | 27 | 30 |
| ≥ 5 peptides | 300 | 137 | 163 |
| In total | 640 | 320 | 320 |

**Table S2**. Distribution of bitter and non-bitter peptides in the four clusters

| **Cluster** | **Total peptides** | **Bitter peptides** |
| --- | --- | --- |
| 1 | 183 | 16 |
| 2 | 196 | 56 |
| 3 | 140 | 129 |
| 4 | 121 | 119 |

**Table S3.** Distribution of Q values within the four clusters regarding their total quantity and their relative percentage with respect to the whole dataset.

|  | **1** | **2** | **3** | **4** |
| --- | --- | --- | --- | --- |
| **Peptides [in total] with Q-Value <1300 cal/mol** | 49 | 55 | 45 | 23 |
| Percentage of peptides [%] with Q-Value <1300 cal/mol | 7.66 | 8.59 | 7.03 | 3.59 |
| Bitter [in total] | 2 | 18 | 44 | 23 |
| Non-bitter [in total] | 47 | 37 | 1 | 0 |
| **Peptides [in total] with Q-Value >1400 cal/mol** | 117 | 128 | 93 | 88 |
| Percentage of peptides [%] with Q-Value >1400 cal/mol | 18.28 | 20.01 | 14.53 | 13.75 |
| Bitter [in total] | 13 | 36 | 84 | 86 |
| Non-bitter [in total] | 104 | 92 | 9 | 2 |
| Undefined [%] between with Q-Value 1300 – 1400 cal/mol | 2.66 | 2.03 | 0.31 | 1.56 |

**Table S4.** Highest and lowest values for Q-value, the number of amino acids containing in oligo- and polypeptides and the molecular weight in the four clusters.

| **Cluster 1** | | |
| --- | --- | --- |
|  | **Min** | **max** |
| MW[Da] | 160 | 1806 |
| Number of amino acids | 2 | 15 |
| Q value [cal/mol] | -5 | 2692 |
| **Cluster 2** | | |
| MW[Da] | 146 | 4297 |
| Number of amino acids | 2 | 39 |
| Q value [cal/mol] | -50 | 2985 |
| **Cluster 3** | | |
| MW[Da] | 132 | 2060 |
| Number of amino acids | 2 | 19 |
| Q value [cal/mol] | 0 | 3000 |
| **Cluster 4** | | |
| MW[Da] | 172 | 1661 |
| Number of amino acids | 2 | 14 |
| Q value [cal/mol] | 365 | 2825 |

**Table S5.** Q-values of amino acids.

| **Amino Acid** | **Δf (cal/mol)** |
| --- | --- |
| Glutamine - Gln - Q | -100 |
| Asparagine - Asn - N | -10 |
| Glycine - Gly - G | 0 |
| Alanine - Ala - A | 730 |
| Valine - Val - V | 1690 |
| Leucine - Leu - L | 2420 |
| Isoleucine - Ile - I | 2970 |
| Phenylalanine - Phe - F | 2650 |
| Proline - Pro - P | 2600 |
| Methionine - Met - M | 1300 |
| WTyrosine - Tyr - Y | 2870 |
| Threonine - Thr - T | 440 |
| Serine - Ser - S | 40 |
| Aspartic acid - Asp - D | 540 |
| Glutamic acid - Glu - E | 550 |
| Arginine - Arg - R | 730 |
| Lysine - Lys - K | 1500 |
| Triptophan - Trp - W | 3000 |

**Tables S6**. Ranked importance scores of extracted substructures from BitterPep-GCN for bitterness (left table) or non-bitterness (right table).


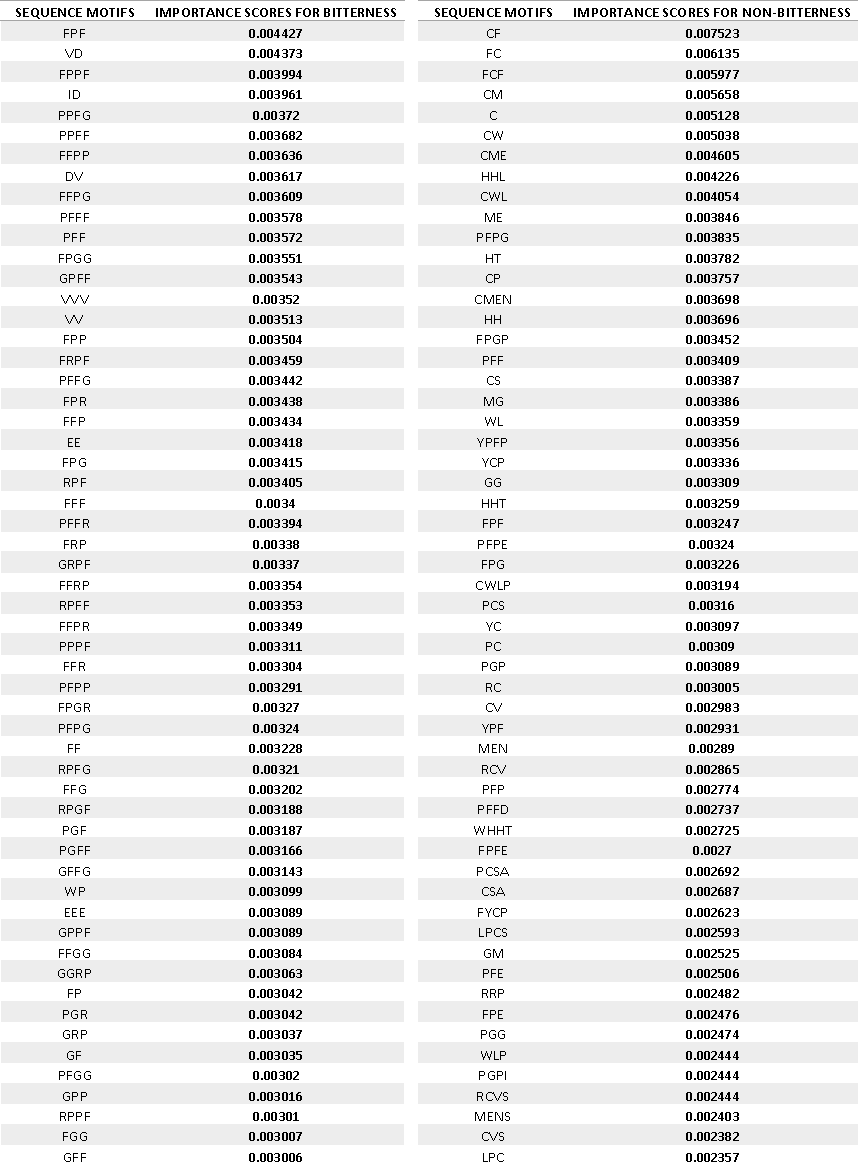


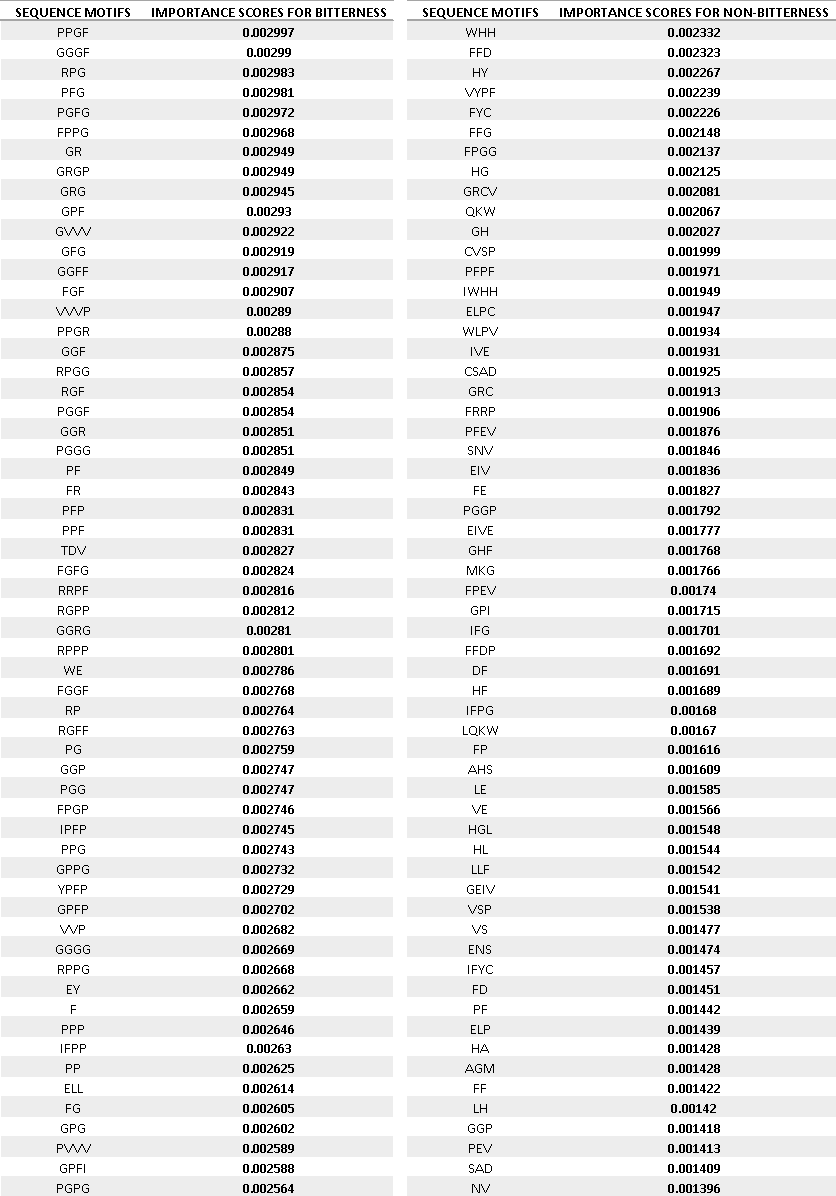


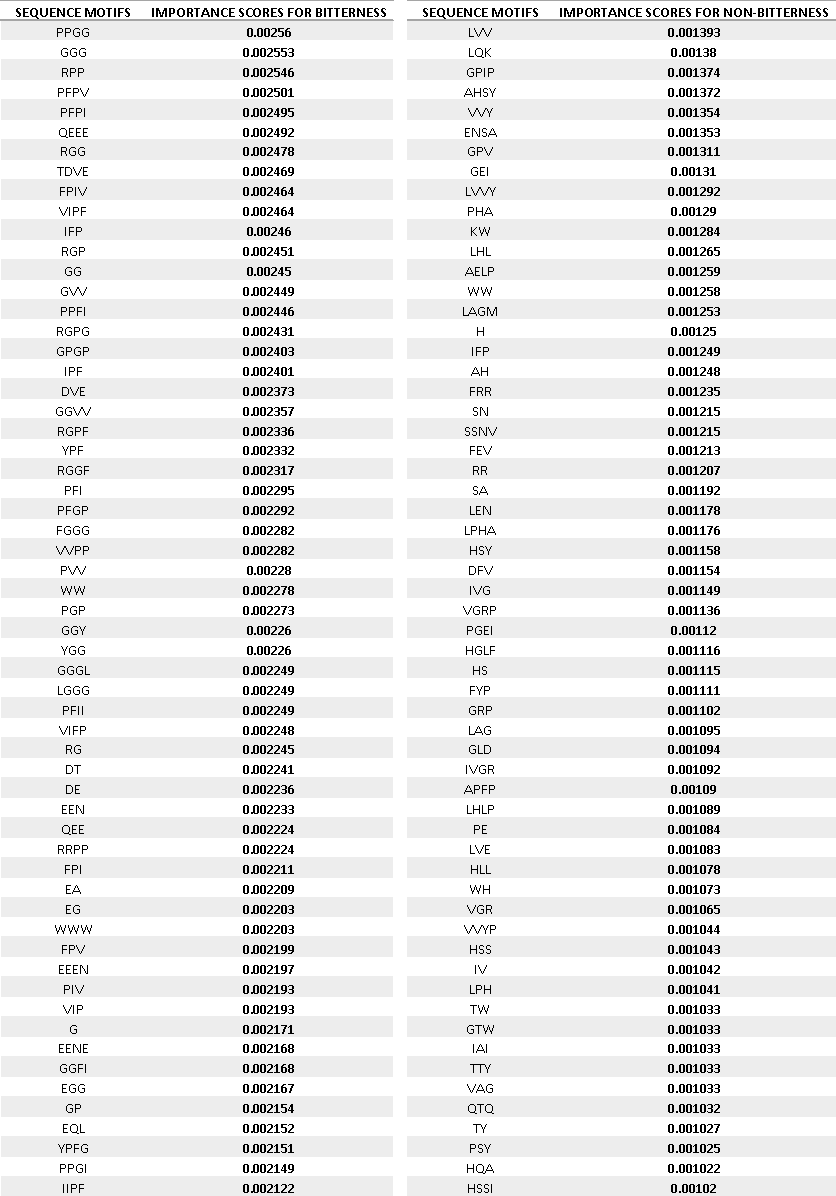


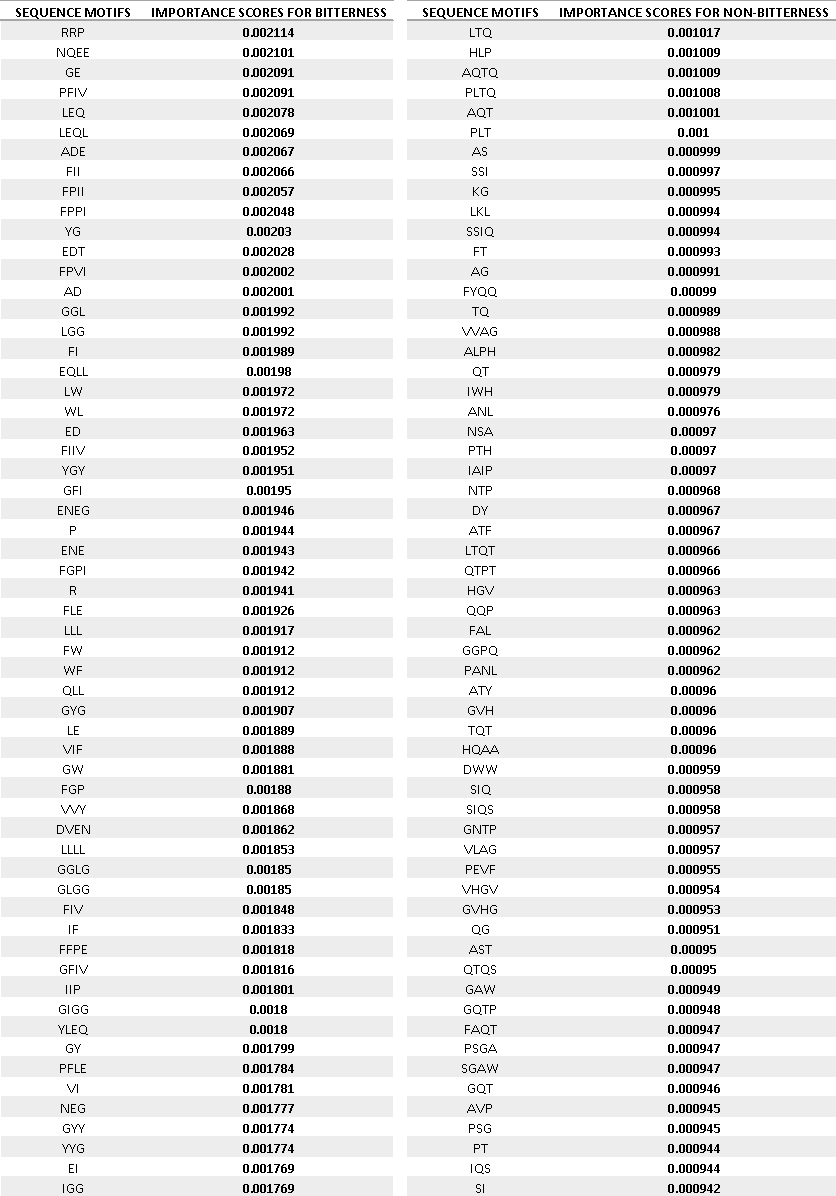


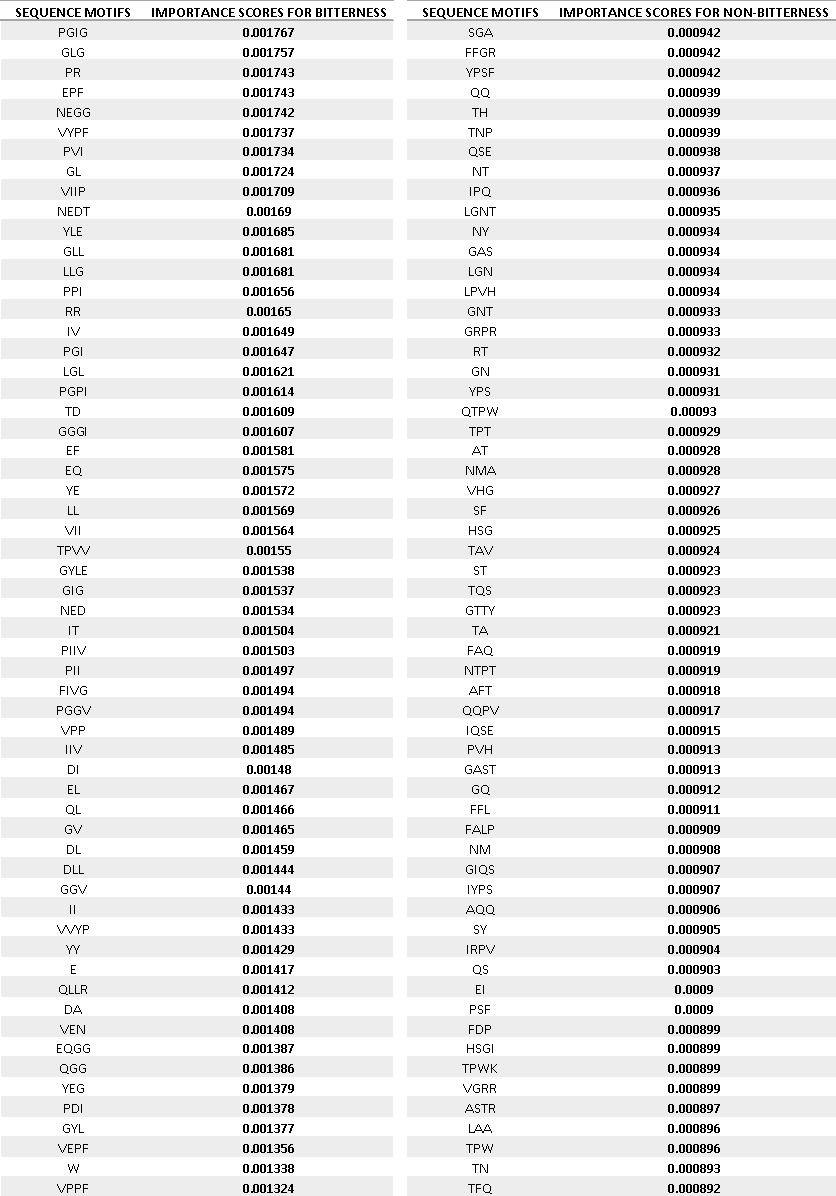


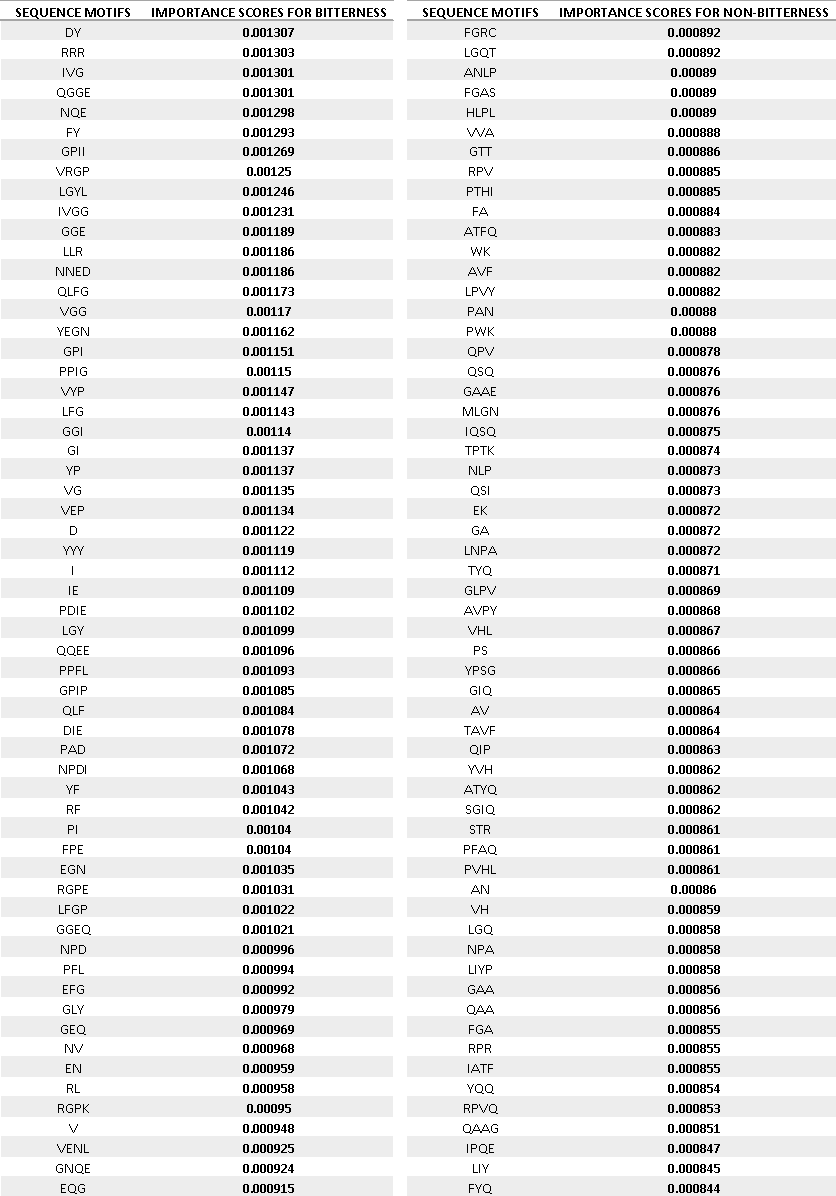


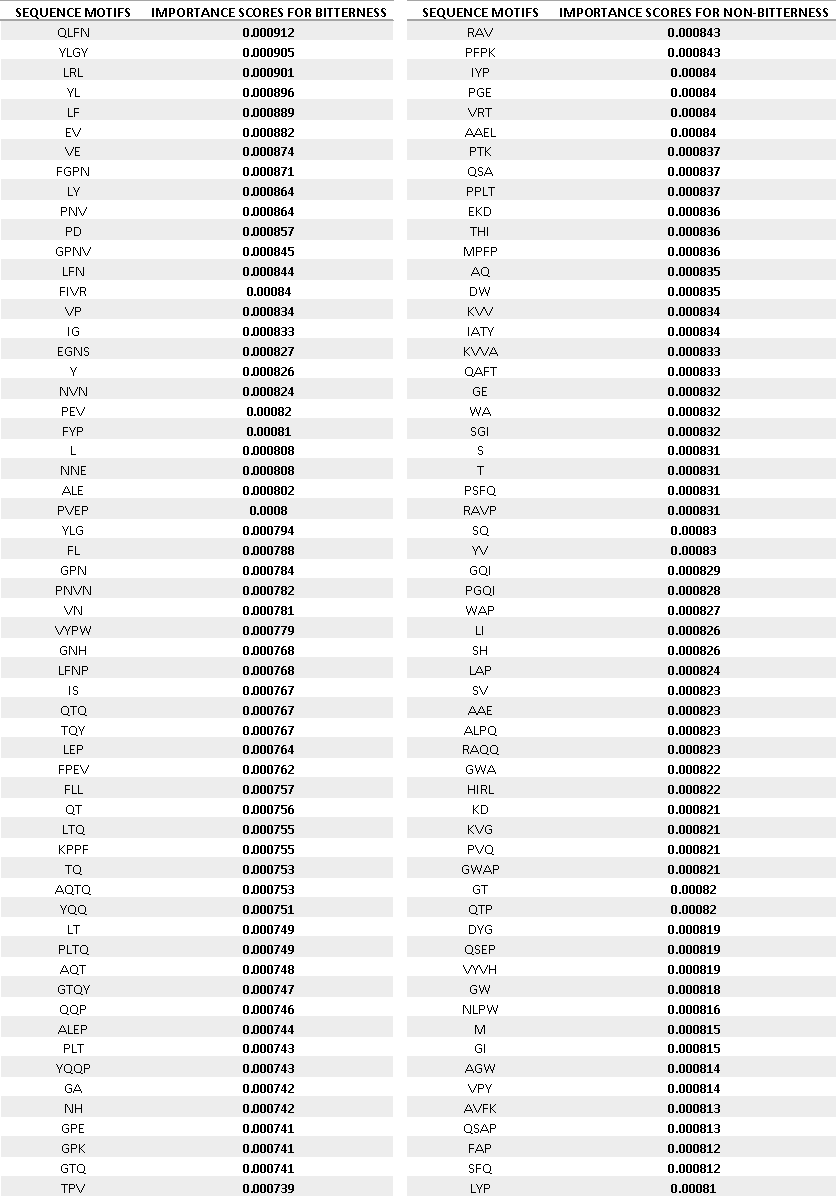


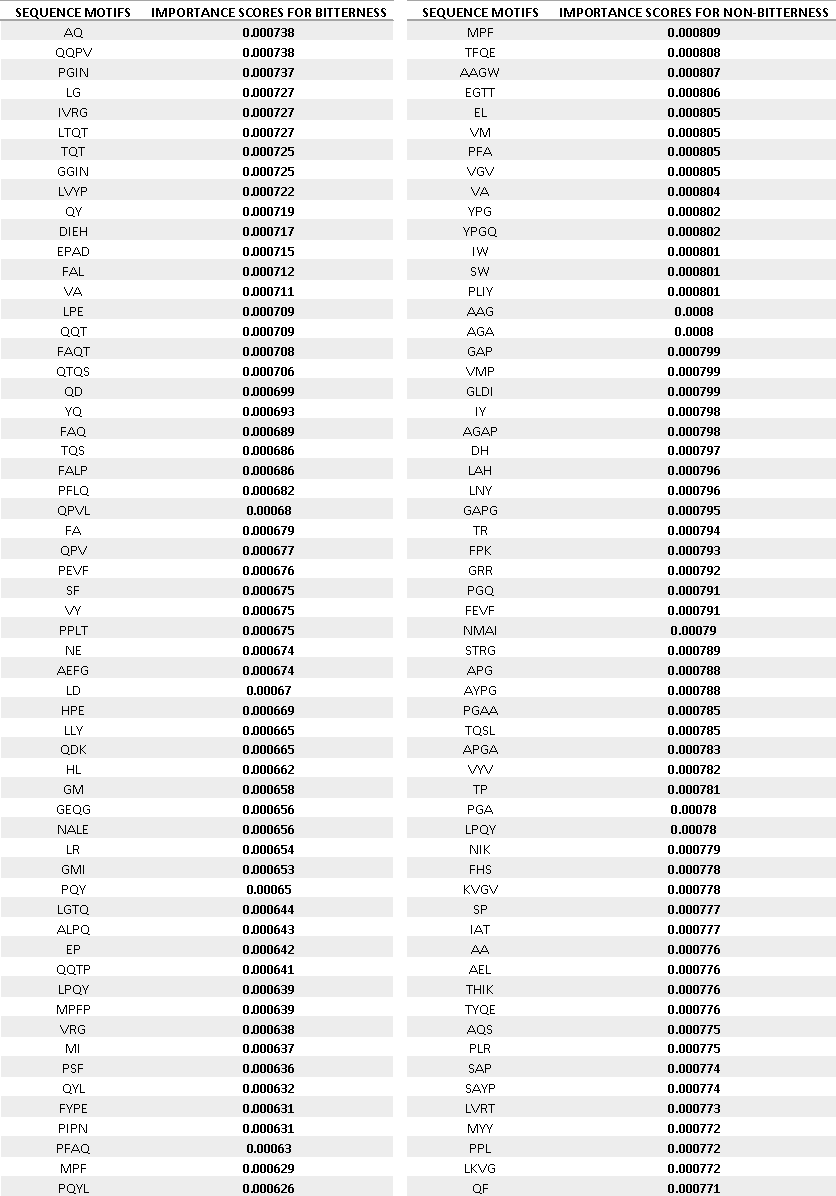


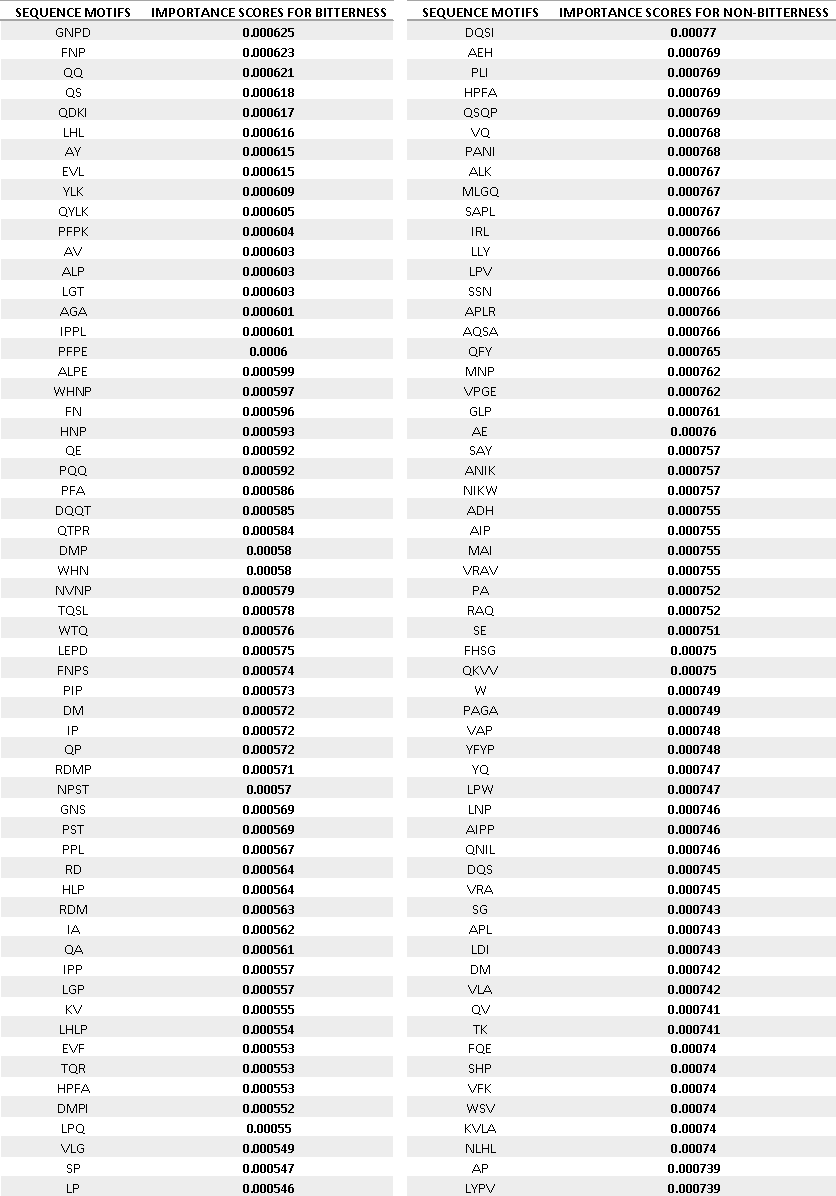


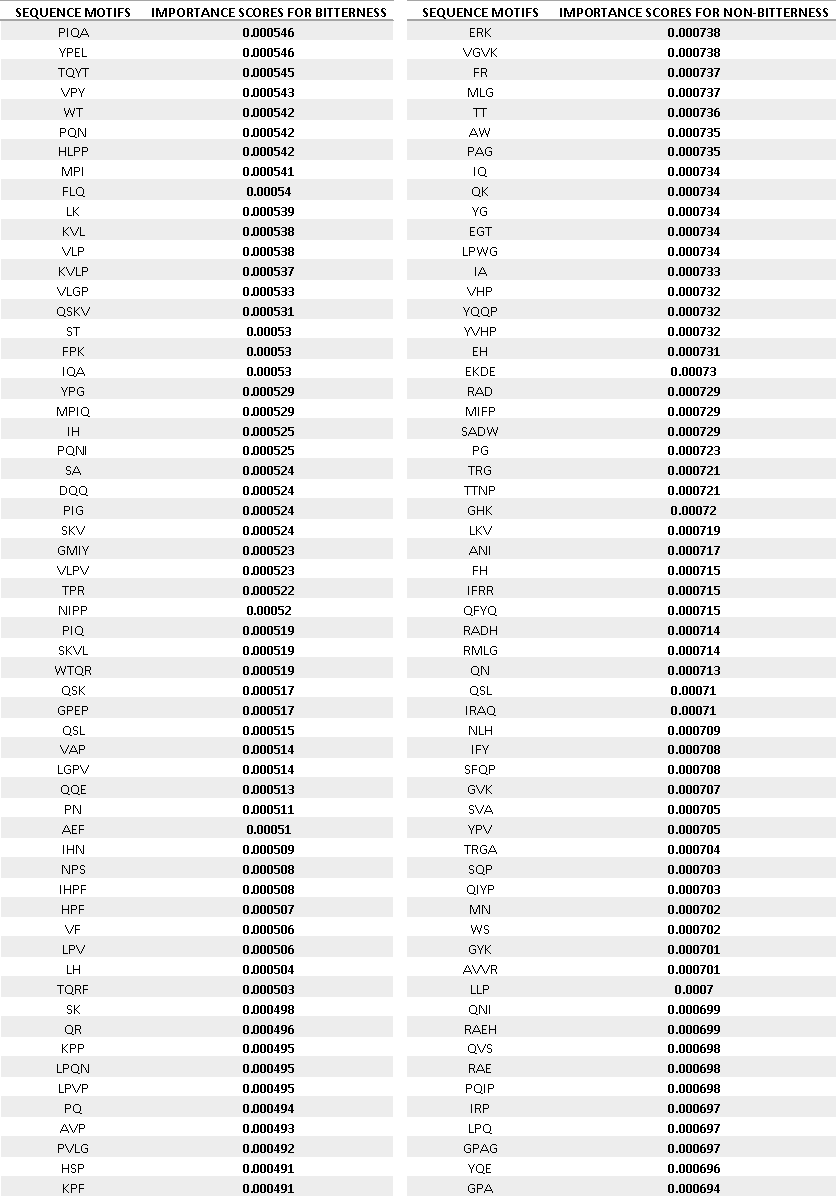


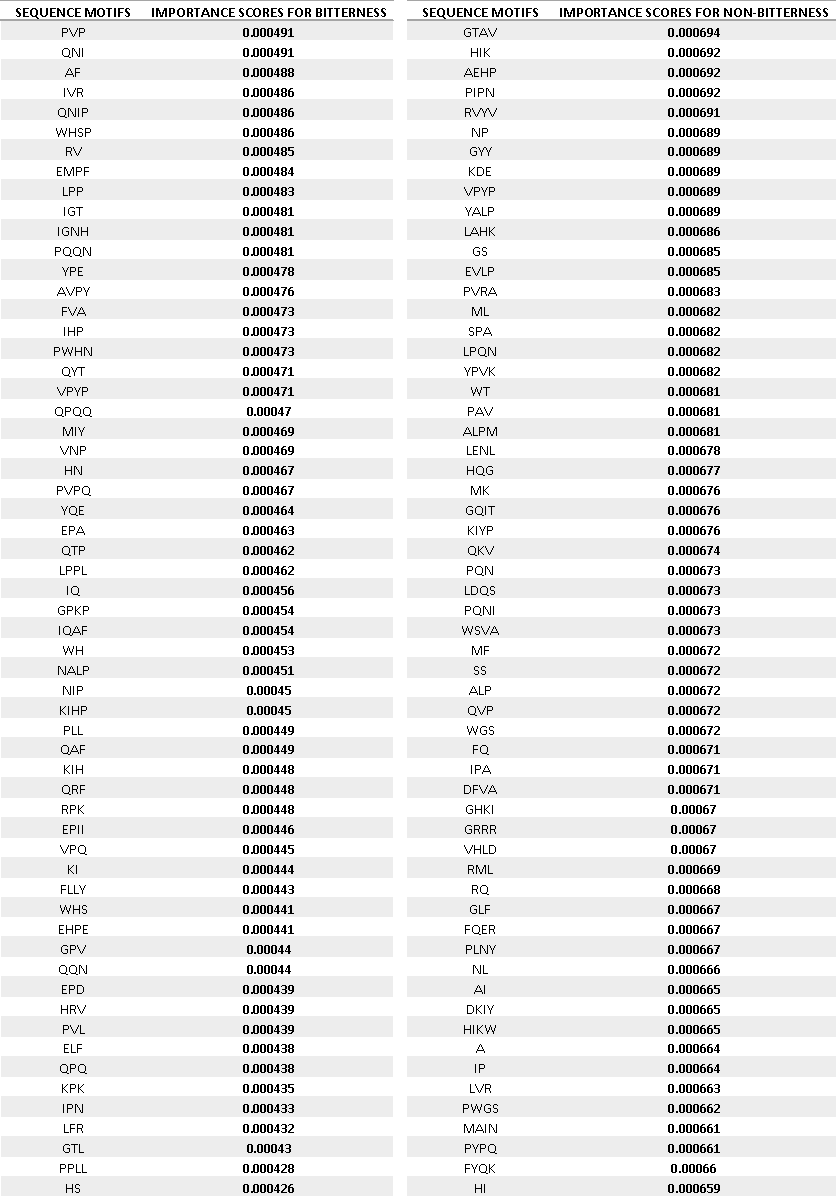


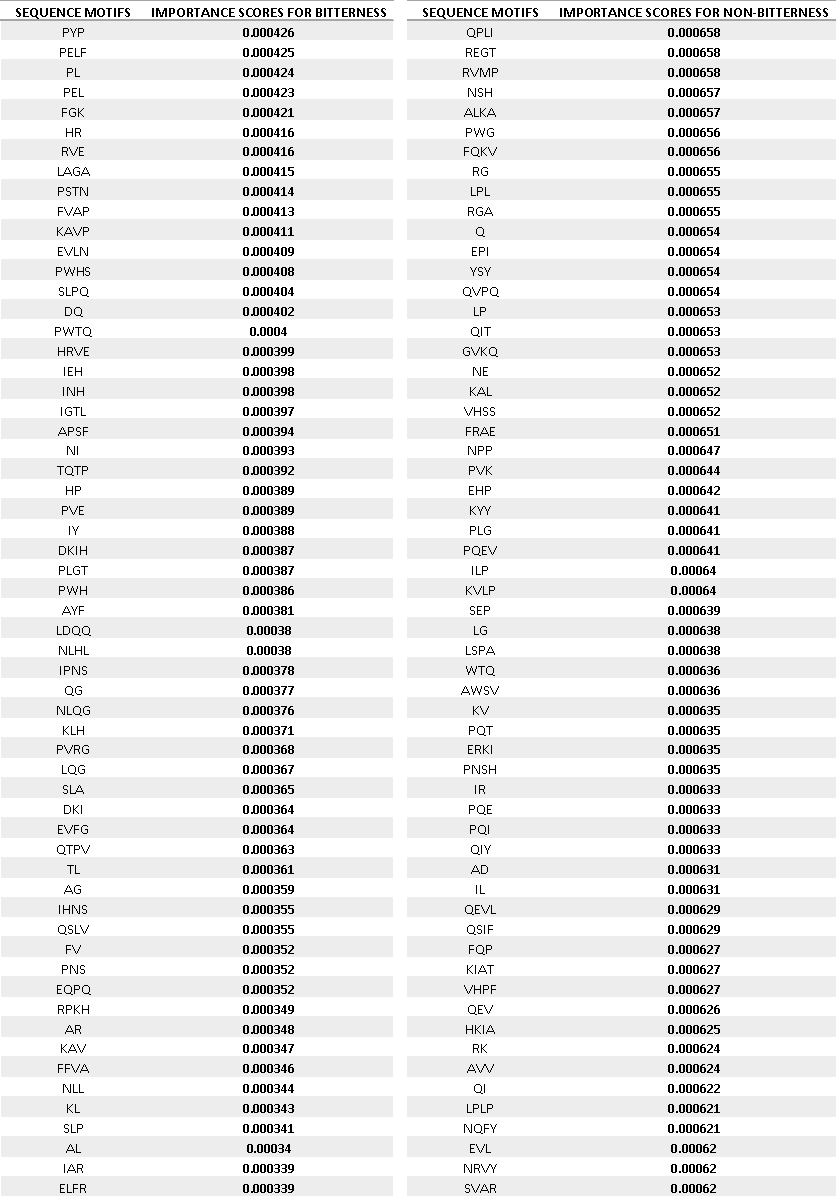


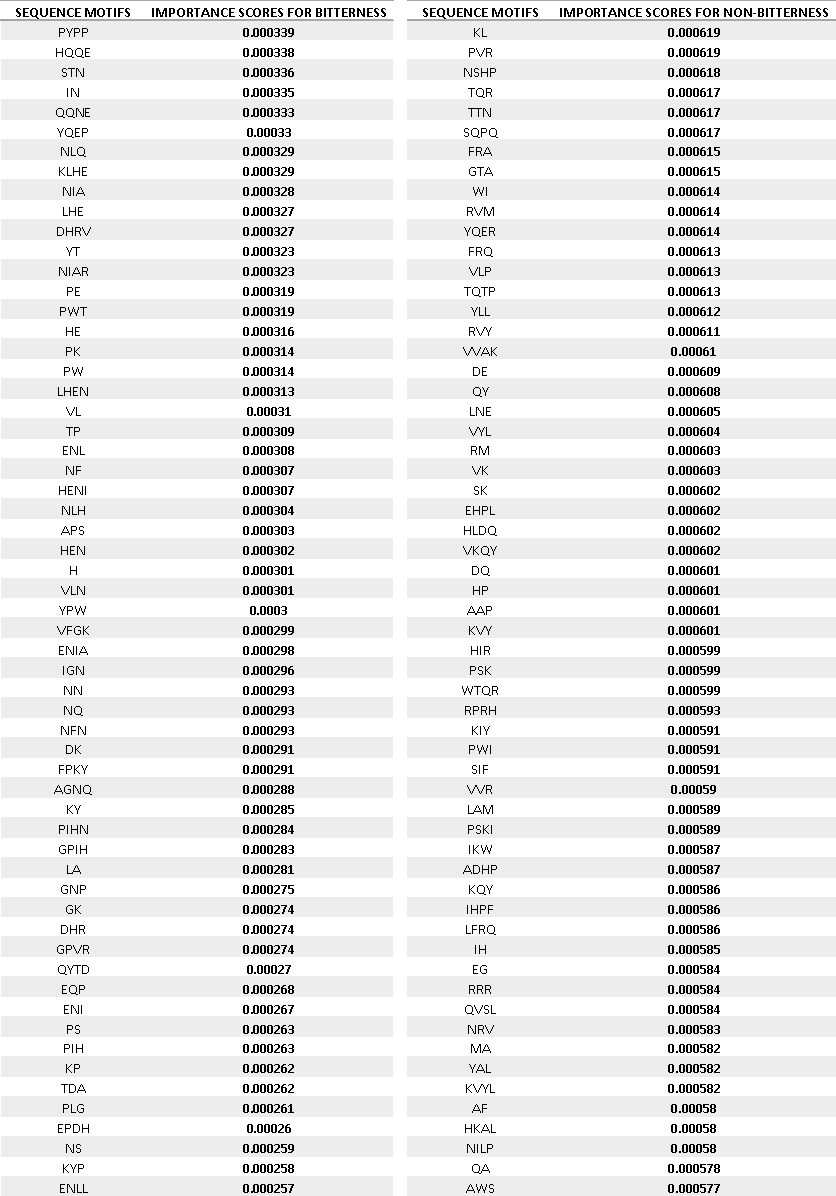


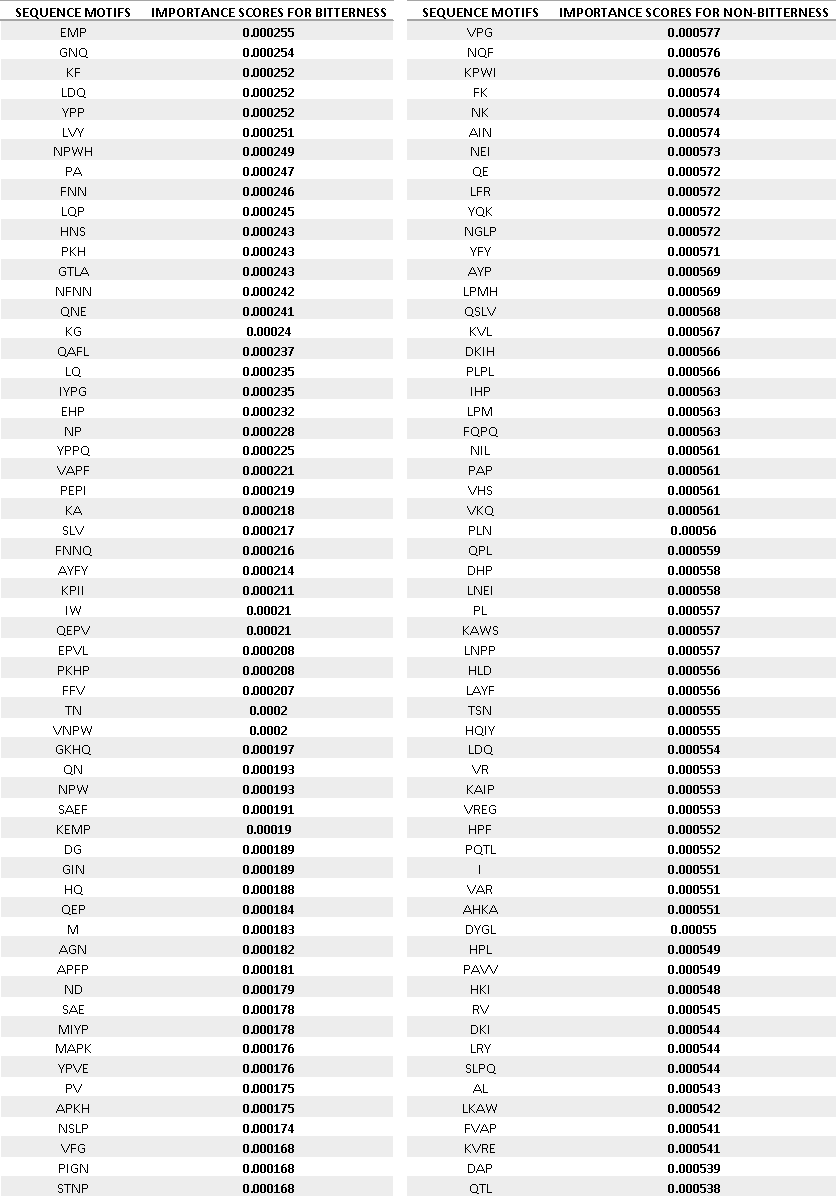


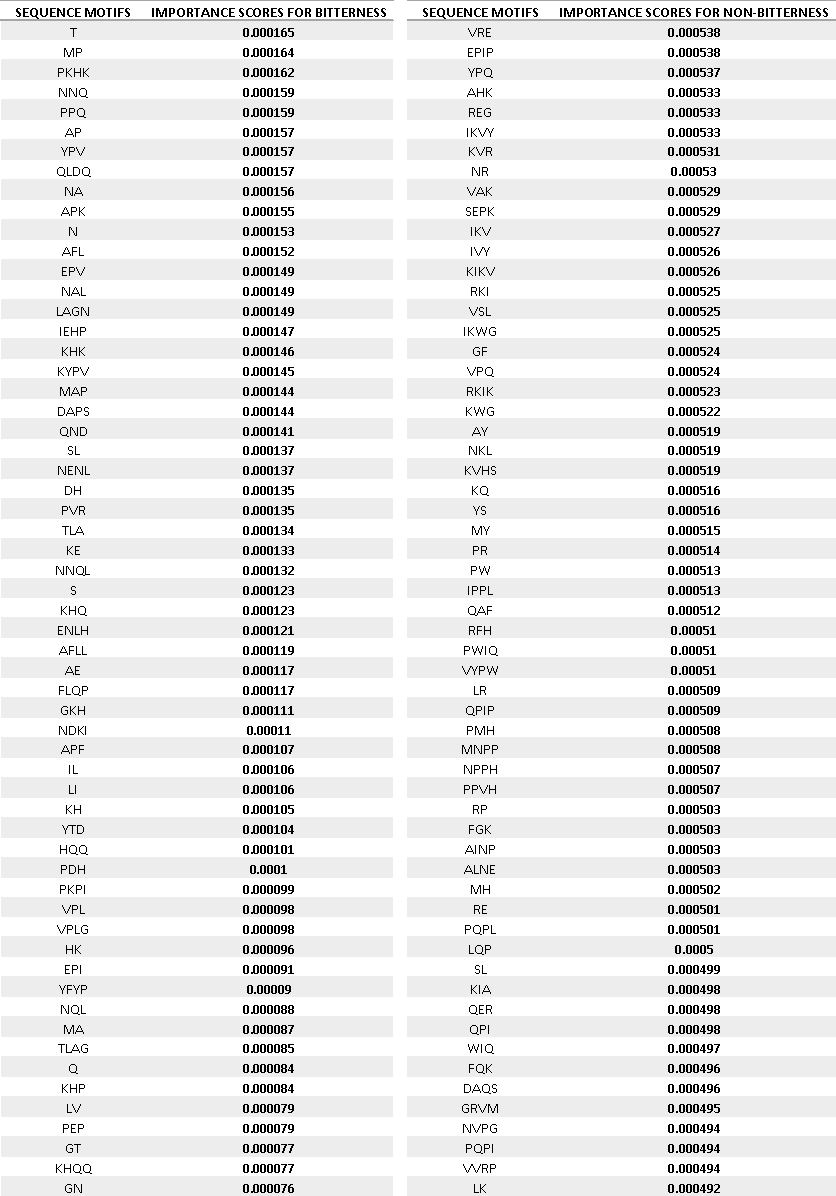


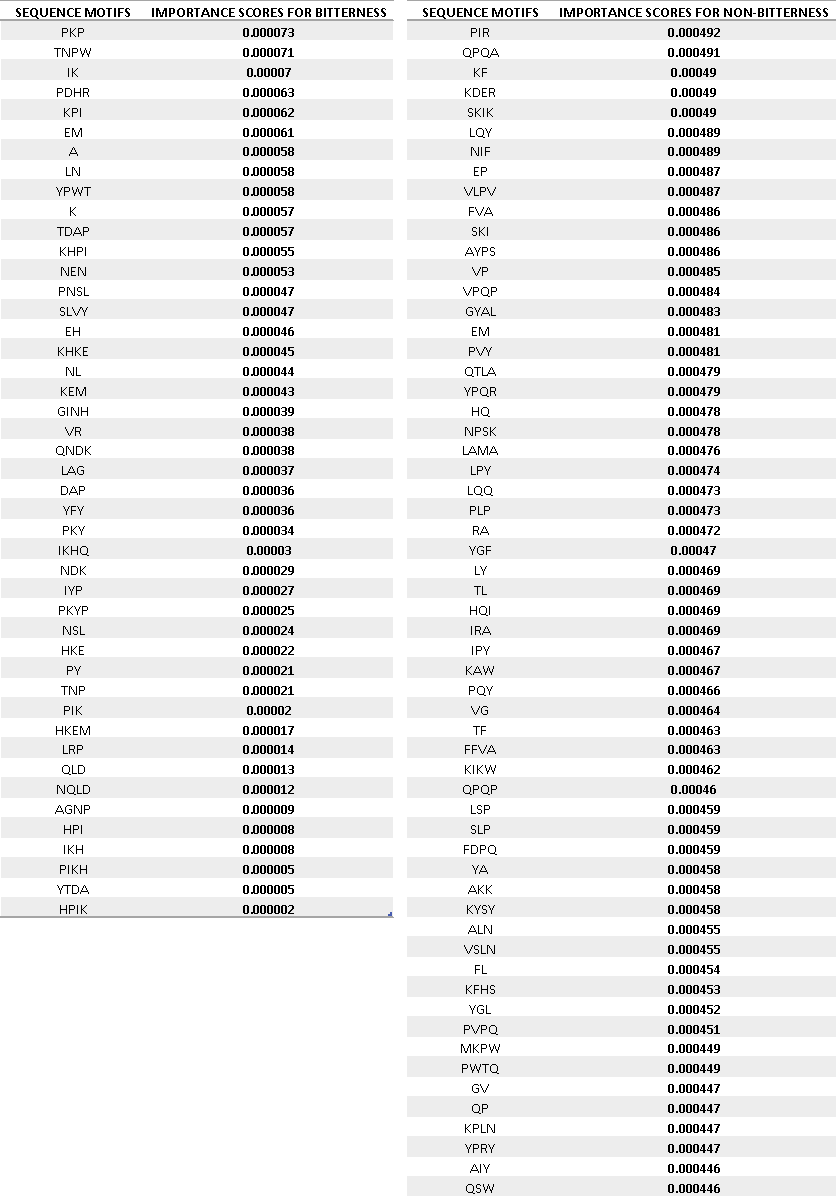


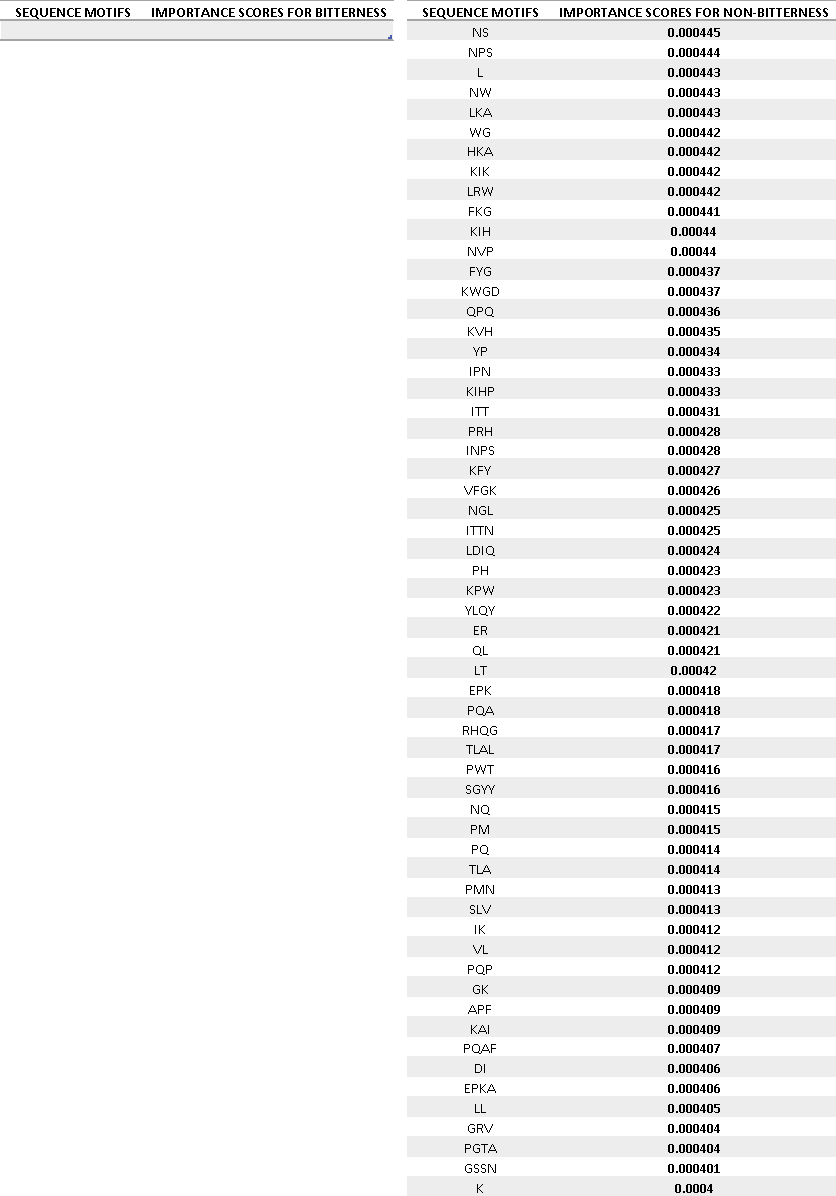


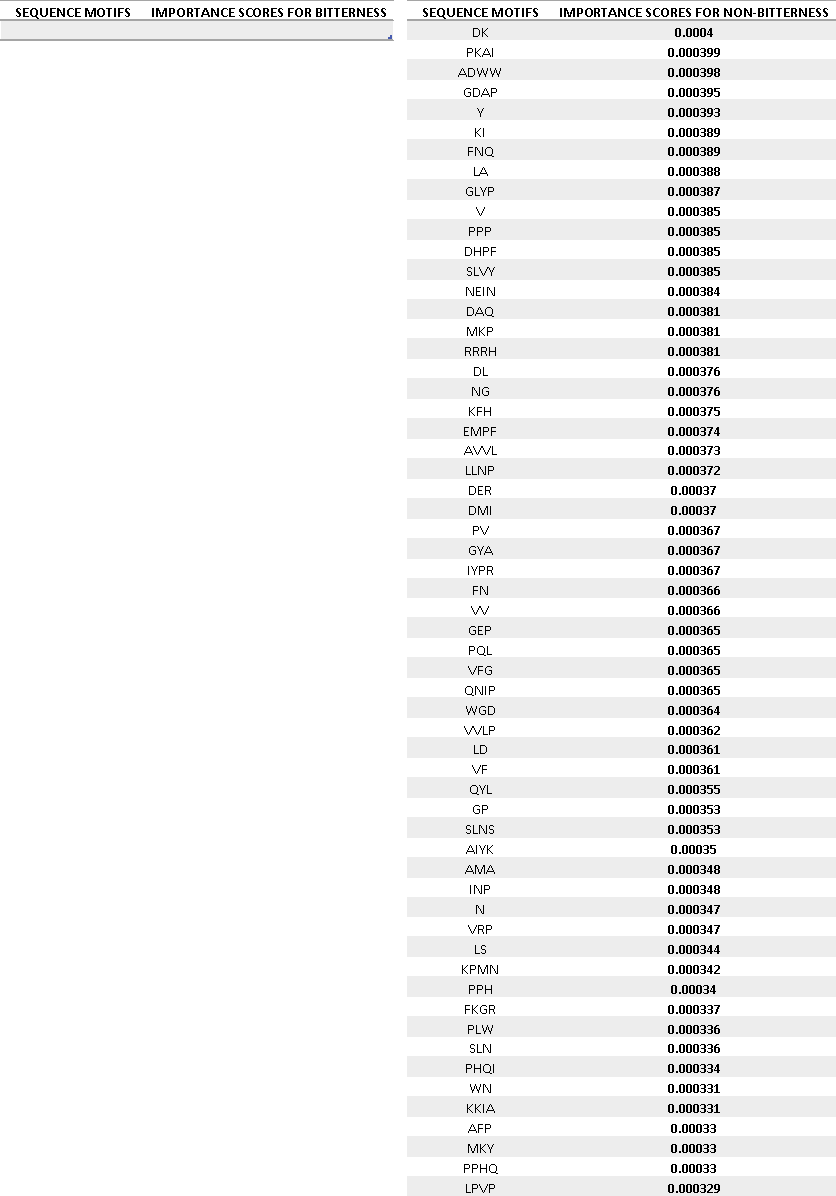


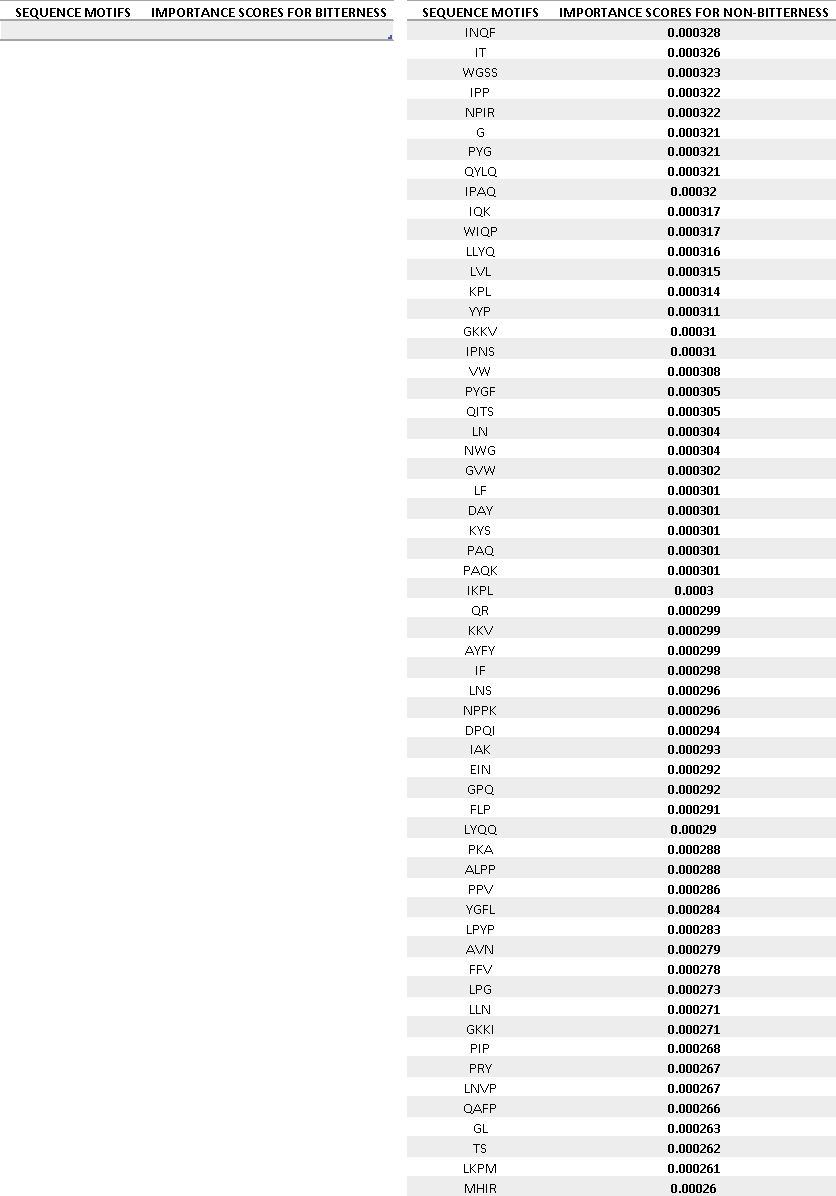


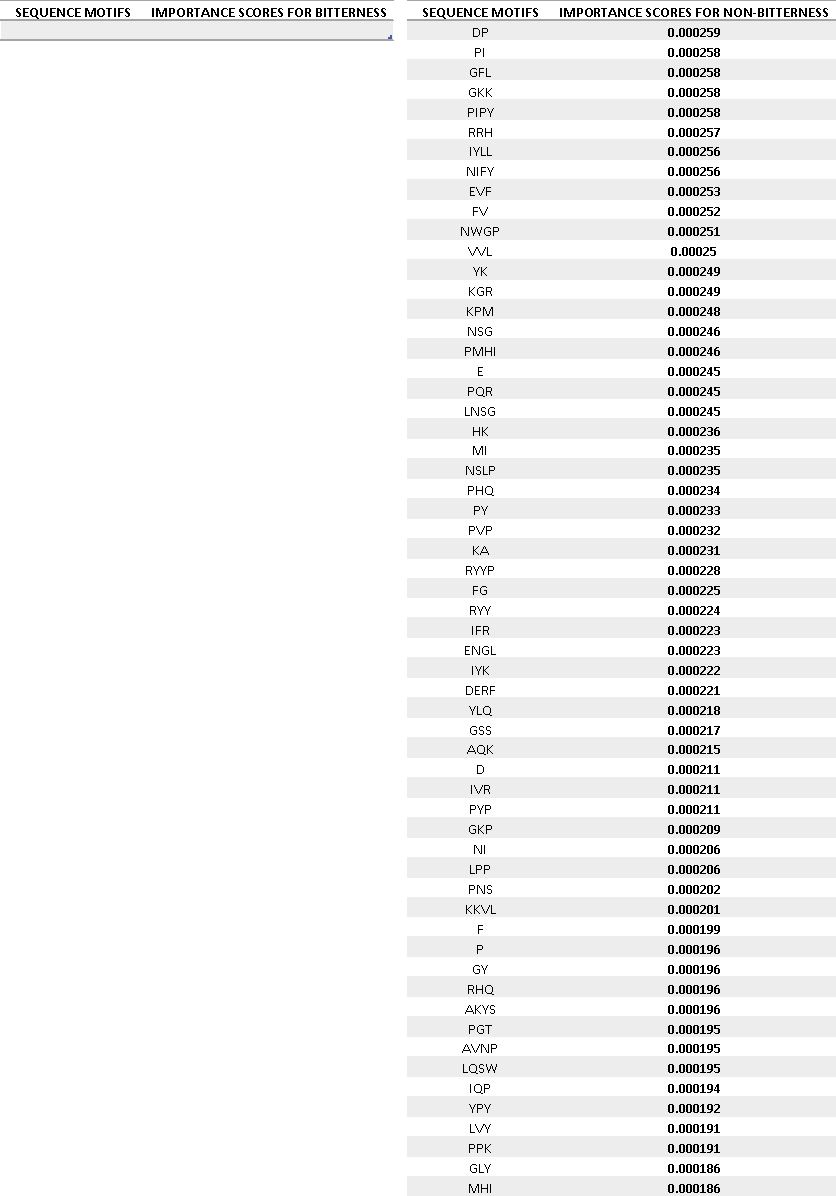


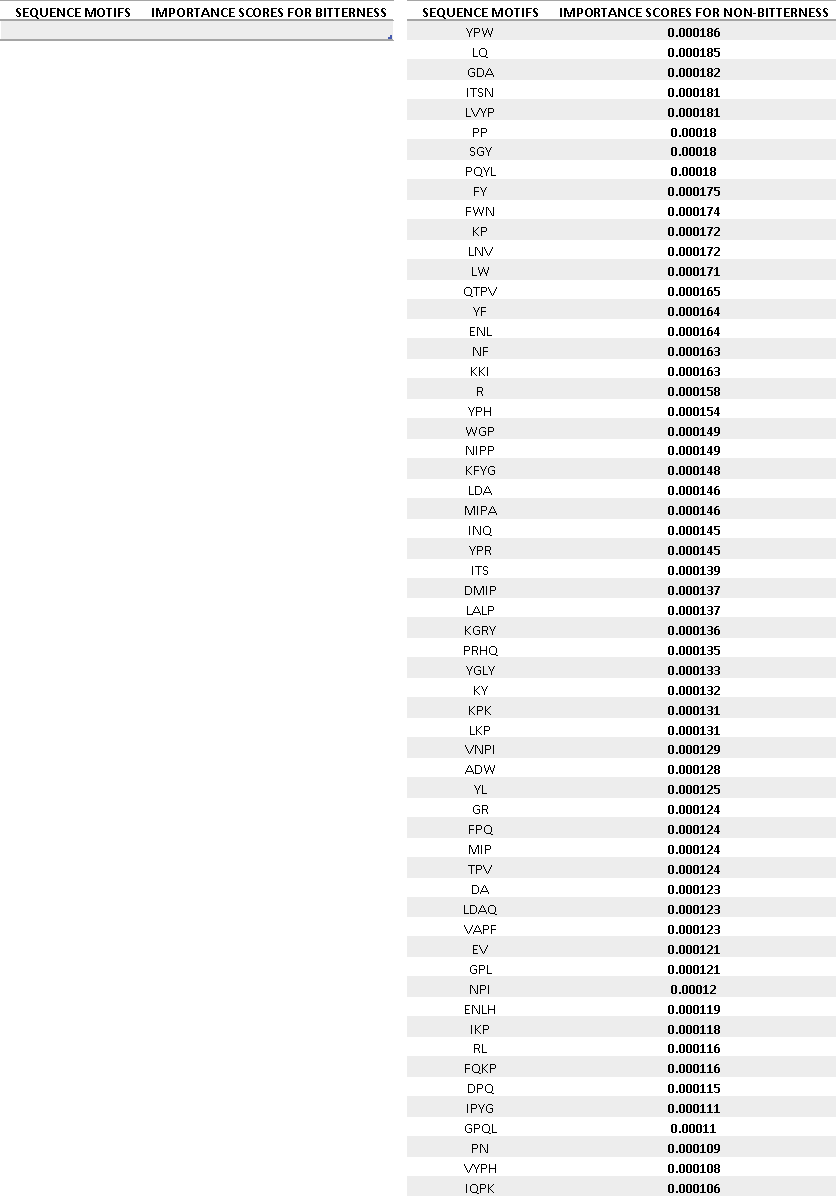


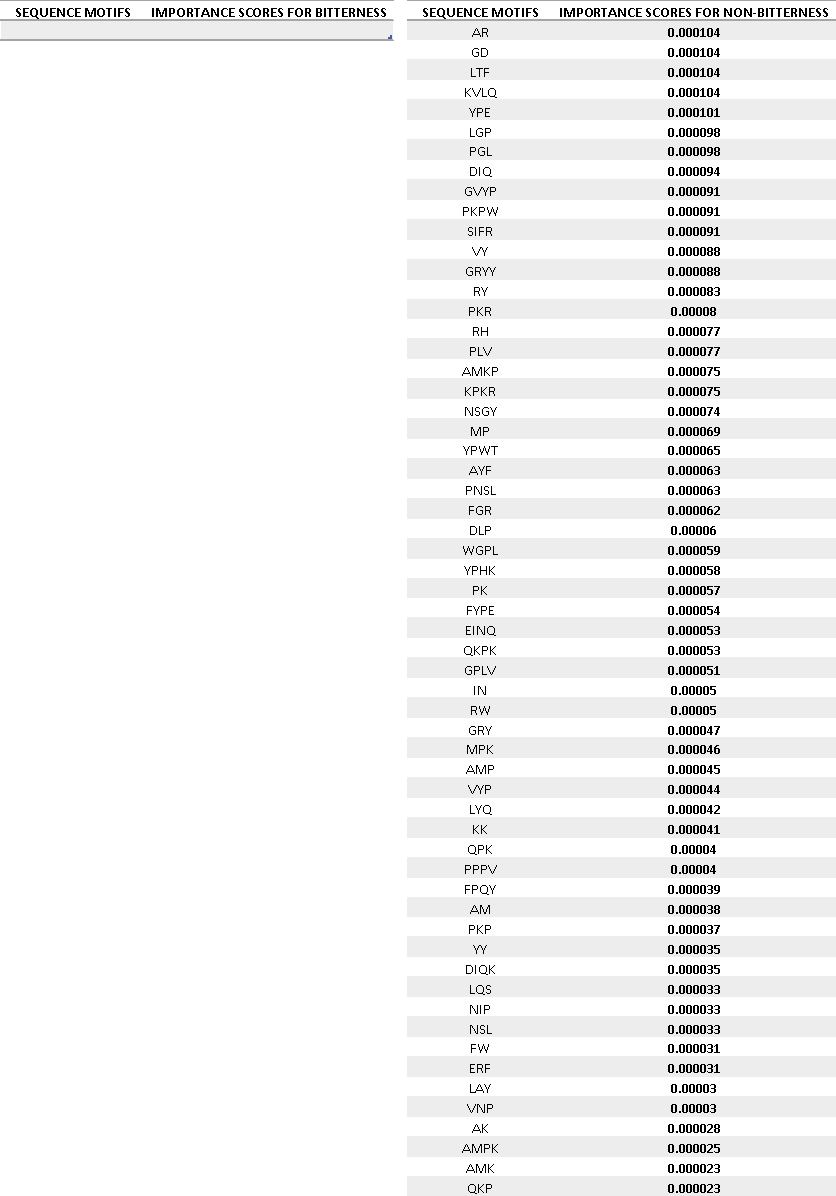


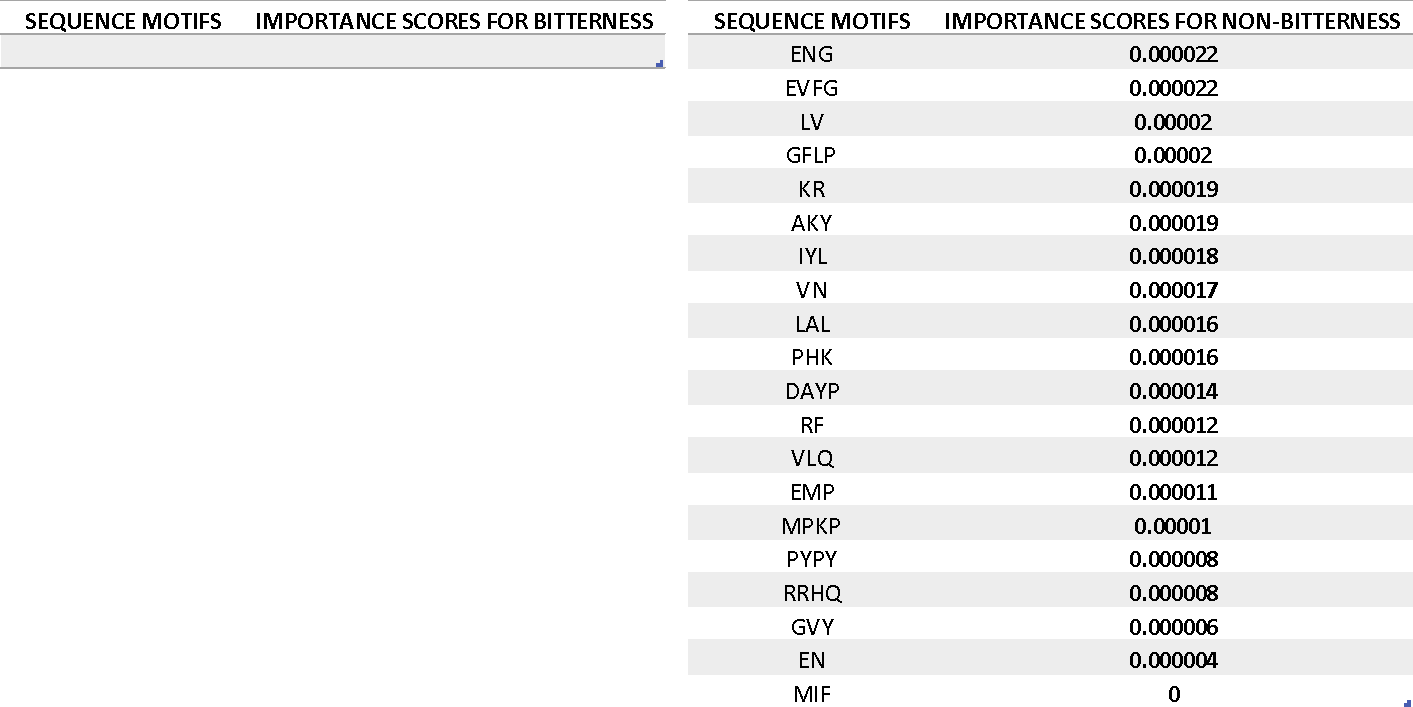


**References**

Charoenkwan, P., J. Yana, N. Schaduangrat, C. Nantasenamat, M. M. Hasan and W. Shoombuatong (2020). "iBitter-SCM: Identification and characterization of bitter peptides using a scoring card method with propensity scores of dipeptides." Genomics **112**(4): 2813-2822.

Dagan-Wiener, A., Nissim, I., Ben Abu, N. et al. Bitter or not? BitterPredict, a tool for predicting taste from chemical structure. Sci Rep 7, 12074 (2017). <https://doi.org/10.1038/s41598-017-12359-7>
